# Selection of suitable reference genes for gene expression studies in myxosporean (Myxozoa, Cnidaria) parasites

**DOI:** 10.1038/s41598-019-51479-0

**Published:** 2019-10-21

**Authors:** Anush Kosakyan, Gema Alama-Bermejo, Pavla Bartošová-Sojková, Ana Born-Torrijos, Radek Šíma, Anna Nenarokova, Edit Eszterbauer, Jerri Bartholomew, Astrid S. Holzer

**Affiliations:** 1 Institute of Parasitology, Biology Centre, Czech Academy of Sciences, 37005, Ceske Budejovice, Czech Republic; 2 Department of Microbiology, Oregon State University, Corvallis, Oregon USA; 3 Centro de Investigación Aplicada y Transferencia Tecnológica en Recursos Marinos Almirante Storni (CIMAS), CCT CONICET – CENPAT, San Antonio Oeste, Argentina; 4 Faculty of Science, University of South Bohemia, Ceske Budejovice, Czech Republic; 5 Institute for Veterinary Medical Research, Centre for Agricultural Research, Hungarian Academy of Sciences, Budapest, Hungary

**Keywords:** Gene expression, Parasite genetics

## Abstract

Myxozoans (Cnidaria: Myxozoa) are an extremely diversified group of endoparasites some of which are causative agents of serious diseases in fish. New methods involving gene expression studies have emerged over the last years to better understand and control myxozoan diseases. Quantitative RT-PCR is the most extensively used approach for gene expression studies. However, the accuracy of the results depends on the normalization of the data to reference genes. We studied the expression of eight commonly used reference genes, adenosylhomocysteinase (AHC1), beta actin (ACTB), eukaryotic translation elongation factor 2 (EF2), glyceraldehyde-3-phosphate dehydrogenase (GAPDH), hypoxanthine-guanine phosphoribosyltransferase 1 (HPRT1), DNA-directed RNA polymerase II (RPB2), 18S ribosomal RNA (18S), 28S ribosomal RNA (28S) across different developmental stages of three myxozoan species, *Sphaerospora molnari*, *Myxobolus cerebralis* and *Ceratonova shasta*, representing the three major myxozoan linages from the largest class Myxosporea. The stable reference genes were identified using four algorithms: geNorm, NormFinder, Bestkeeper and Δ*Cq* method. Additionally, we analyzed transcriptomic data from *S. molnari* proliferative and spore-forming stages to compare the relative amount of expressed transcripts with the most stable reference genes suggested by RT-qPCR. Our results revealed that GAPDH and EF2 are the most uniformly expressed genes across the different developmental stages of the studied myxozoan species.

## Introduction

Myxozoans are a cnidarian group of obligate parasites documented mainly from fish in marine and freshwater habitats. These microscopic endoparasites have a two-host life cycle that involves an invertebrate (annelids and bryozoans) and a vertebrate host (mostly fish, few are known from other vertebrates) where infectious actinospores and myxospores are formed, respectively, serving as transmission stages in aquatic habitats^1,2^. The current classification of the Myxozoa into classes mainly reflects spore morphology and invertebrate host types. Taxa are ranked in the class Myxosporea Bütschli 1881 according to their hardened shell valves and annelid definitive hosts while the ones with soft spore valves and bryozoan definitive hosts are representatives of the second class Malacosporea Canning, Curry, Feist, Longshaw et Okamura 2000. Myxosporea represents the largest class comprising 19 families and 67 genera while Malacasporea have only one family and two genera^3^.

Myxosporeans have received considerable attention since some of them are reported to cause severe fish diseases. These parasites can have a strong impact on wild and cultured fish worldwide by reducing fillet marketability and causing important mortalities in fish populations^4–8^. Given the fact that aquaculture is one of the fastest growing food sectors^9,10^ comprising an ample part of global food production, economic losses caused by parasites such as myxosporeans are of major concern^11,12^. Furthermore, disease severity has been linked to increasing water temperatures (i.e. in *Ceratonova shasta*^13,14^), predicting emerging numbers of these organisms in the future as a result of climate change.

In this study we have focused on three myxosporean species, *Sphaerospora molnari*, *Myxobolus cerebralis* and *Ceratonova shasta* that belong to three (sphaerosporids, oligochaete-infecting and polychaete-infecting lineages^15^) out of four main myxozoan phylogenetic linages and transcriptomic data are available (^16^, Alama-Bermejo *et al*. submitted, Hartigan *et al*. submitted). These species are considered serious pathogens for highly commercialized fishes such as cyprinids and salmonids which represent a significant proportion of worldwide aquaculture production. *Sphaerospora molnari* causes respiratory and osmoregulatory failure in host’s gill epithelia^17^, while proliferative blood stages induce a massive systemic inflammatory response^18^. It was also shown that *S. molnari* may be an important co-factor for swim bladder inflammation in carp, the disease responsible for up to 100% mortalities of carp fingerling stocks in Central Europe^19^. The invertebrate definitive host of *S. molnari* is unknown. *Myxobolus cerebralis* causes serious damage to farmed and wild salmonid fish populations worldwide. It is responsible for whirling disease, a condition caused by infection of the hosts central nervous system and cartilage resulting high mortalities^6^. The life cycle of *M. cerebralis* involves the oligochaete *Tubifex tubifex* and salmonids as vertebrate hosts. *Ceratonova shasta* is a serious pathogen of wild and cultured salmonids in the Pacific Northwest of North America, including endangered Coho and Chinook salmons. *C. shasta* causes intestinal enteritis with up to 100% mortality in certain populations. The definitive invertebrate host of *C. shasta* is the freshwater polychaete *Manayunkia speciosa*^20^.

There are currently no disease control methods for myxozoans in general, as no vaccines or commercial treatments for fish destined for human consumption are available. In order to design efficient methods for prediction and control of myxozoan diseases it is important to explore genes that these parasites use for invasion of and survival within their host, e.g. genes involved in immune evasion, since they are potential candidates for targeted antiparasitic treatments and vaccine development. As a result of this need, the number of gene expression studies are presently expanding considerably in order to predict and control such functional genes.

RT-qPCR (reverse transcriptase quantitative PCR) is one of the most rapidly incorporated techniques in scientific studies. Its application in mRNA quantification has grown from ~8% to ~73–88% in the last decade^21^.

Being considered highly sensitive, RT-qPCR is one of the most extensively used approaches for gene expression studies in all organisms^22–24^. To achieve accurate gene expression results, it is critical that RT-qPCR results are normalized to an internal control, since gene expression can be influenced by different factors, i.e. variation in the amount of starting material, differences in RNA contents between cells or developmental stages, technical variability, and transcription efficiency. Traditionally, housekeeping genes (hereafter HKGs) are used as internal control. HKGs are present in all cell types because they are necessary for basic cell survival. HKGs commonly used as internal controls include beta actin, glyceraldehyde-3-phosphate dehydrogenase, several ribosomal genes such as 18S rRNA, 28S rRNA and eukaryotic elongation factors. Due to their key roles in metabolism, cytoskeleton and ribosome structure, the mRNA/rRNA synthesis of these genes was considered to be stable or uniformly expressed in various tissues, during ontology and development, even under different treatments^19–21^ and thus these genes were considered good reference genes (hereafter RGs).

However, it was shown that HKGs independent of organism do not always perform as good RG, and their expression may be differentially regulated and vary under certain experimental conditions. That is why it is highly recommended to validate the HKGs for each organism and study before performing gene expression studies^23^.

For cnidarians in general, data on RGs are scarce, although few differential expression studies were performed^25–30^. For myxozoans, comprehensive gene expression studies are inexistent and only three reports study parasite gene expression^31–33^ and mainly rely on RGs that were “used in previous publications” (e.g.^31^), or the validation of RGs was focused on a limited part of parasite development (e.g. early intrapiscine development studied by Eszterbauer *et al*.^32^). Myxozoans are some of the oldest metazoan parasites with an extremely accelerated evolutionary rate and high heterogeneity across genes^15^, and likely functional derivation of genes. Therefore, it can be expected that genes that serve as RGs in other organisms are not constantly expressed during the complex life cycle of myxozoans and across the different developmental stages, within the vertebrate and invertebrate hosts. 18S rDNA is presently the most commonly used gene region for phylogenetic studies^15^ and especially for PCR and qPCR based detection and quantification assays (e.g.^34–36^), since rDNA occurs in tandem repeats and multiple copies in the genome, however it has not previously been tested as a RG. Considering the increasing need to understand and evaluate gene expression in myxozoans, our aim was to investigate the suitability of candidate reference genes in different developmental stages of three myxozoan species. Furthermore, we want to propose “optimal” reference genes that can be used in future myxozoan gene expression studies aimed at the discovery of functional target proteins to control emerging myxozoan diseases.

## Material and Methods

### Parasite collection

For each species different developmental stages (*S. molnari*) and different life cycle stages (*M. cerebralis* and *C. shasta*) were isolated from fish and definitive worm hosts (Table 1).

**Table 1:** Sampling details of selected parasites across different developmental stages and non- infected host used as control.

Parasite species	Host species	Origine of samples	Parasite developmental stage	Host tissue	Number of samples
*Sphaerospora molnari*	*Cyprinus carpio*	Malá Outrata pond, CZ	presporogonic blood stage	blood	5
	*Cyprinus carpio*	Malá Outrata pond, CZ	non infected	blood	2
*Sphaerospora molnari*	*Cyprinus carpio*	Szarvas, HU	sporogonic stage	gills	5
	*Cyprinus carpio*	Szarvas, HU	non infected	gills	2
*Myxobolus cerebralis*	*Oncorhynchus mykiss*	Inst for Veterinary Med Res, Budapest, HU	sporogonic stage	cartilage (head)	4
	*Oncorhynchus mykiss*	Inst for Veterinary Med Res, Budapest, HU	non infected	cartilage (head)	2
*Myxobolus cerebralis*	*Tubifex tubifex*	Inst for Veterinary Med Res, Budapest, HU	triactinomyxon stage	whole worm	4
	*Tubifex tubifex*	Inst for Veterinary Med Res, Budapest, HU	non infected	whole worm	2
*Ceratonova shasta*	*Oncorhynchus mykiss*	Roaring River Hatchery, Scio, OR, USA	mix of presporogonic and sporogonic stages	intestine	3
	*Oncorhynchus mykiss*	Roaring River Hatchery, Scio, OR, USA	non infected	intestine	2
*Ceratonova shasta*	*Oncorhynchus mykiss*	Roaring River Hatchery, Scio, OR, USA	mix of presporogonic and sporogonic stages	ascites	3
	*Oncorhynchus mykiss*	Roaring River Hatchery, Scio, OR, USA	non infected	ascites	2
*Ceratonova shasta*	*Manayunkia sp*.	Fryer Aquatic Animal Health Lab (OSU), USA	tetractinomyxon stage	whole worm	3
	*Manayunkia sp*.	Fryer Aquatic Animal Health Lab (OSU), USA	tetractinomyxon stage	whole worm	2

*S. molnari* proliferative, presporogonic blood stages were collected from a laboratory line that has been cycled (2 + years) from fish to fish by intraperitoneal injection of parasites into specific parasite-free (SPF) common carp (*Cyprinus carpio*) (methodology detailed in^18^). *S. molnari* blood stages (n = 5 fish) were concentrated and co-isolated with host white blood cells from whole blood of carp, by centrifugation for 5 minutes at 3500 rpm in heparinized hematocrit tubes^18^. Spore-forming stages (infected gills, n = 5) were obtained from carp held at the recirculation system of the Research Institute for Fisheries and Aquaculture (Szarvas, Hungary).

*M. cerebralis* actinospores used for exposure trials originated from *Tubifex tubifex* cultures maintained in the laboratory of the Institute for Veterinary Medical Research (IVMR), Budapest, Hungary, over several years. SPF rainbow trout, *Onchorhynchus mykiss* (Kamloops strain) was obtained from the Lillafüred Trout Hatchery, Hungary (yolk sac stage) and reared at the IVMR. Fish were infected individually with 5000 freshly filtered actinospores according to^37^. From infected fish, pieces of skulls containing myxospores and sporogonic plasmodia (spore-forming stages) were collected 90 days’ post exposure (n = 4). Laboratory *T. tubifex* cultures were exposed with spores isolated from the head cartilage as per^38^. Worms (n = 4) infected with triactinomyxon spore-forming stages were collected 100 days’ post exposure.

The species composition of naive worm cultures was regularly checked by DNA sequencing and microscopy, and worm specimens with long hair chaetae (which all belong to *Tubifex tubifex* s.l. in the culture) were selected for individual exposure.

*Ceratonova shasta* was collected from ascitic fluid of the abdominal cavity and from infected intestines of rainbow trout infected with genotype IIR (n = 3). Naive rainbow trout were from Roaring River Hatchery strain (Scio, OR, Oregon Department of Fish and Wildlife) and they were infected by an intraperitoneal injection of ascites collected from an infected rainbow trout that was previously exposed in the Williamson River, Oregon, USA. Fish were held at 18 °C in 100 L tanks at the Aquatic Animal Health Laboratory at Oregon State University (AAHL, OSU). Fish were sampled when developing typical clinical signs of enteronecrosis^6^. A wet mount of ascites was examined using a Zeiss 47 30 28 light microscope and the presence of different developmental stages (plasmodia and spores) was confirmed. Genotype was confirmed using the ITS rDNA region^39^. Fish were euthanized by an overdose of buffered MS-222 (tricaine methanesulfonate; Argent Laboratories). Ascites was collected with a sterile syringe. Intestine was removed by dissection. Fluid and tissues were flash frozen in liquid nitrogen and kept at −80 °C. The infection had been achieved by transmission of ascites stages from fish to fish by intraperitoneal injection^40^.

*Manayunkia* sp. worms (n = 3) infected with actinospores of the same genotype were obtained from laboratory cultures (methodology of^41^ at the John L. Fryer Aquatic Animal Health Lab (OSU). Worms originated from the Upper Klamath River and were regularly seeded with myxospores from IIR transfected rainbow trout^42^. RNA in blood, gills,  skull pieces and worms was stabilized in 100 µl of RNAprotect Cell Reagent (Qiagen) and stored at −80 °C prior to RNA extraction. Intestine and ascites infected with *C. shasta* were flash-frozen in liquid nitrogen and stored at −80 °C.

### Ethics statement

Fish manipulation and sampling techniques were performed in accordance with Czech legislation (Protection of Animals Against Cruelty Act No. 246/1992) and approved by the Czech Ministry of Agriculture. Rainbow trout sampling at Oregon State University (OSU) was carried out in accordance with the recommendations of OSU - Institutional Animal Care and Use Committee (IACUC). The protocol was approved by ACUP #4666. For rainbow trout exposure to *M. cerebralis*, the Hungarian Scientific Ethical Committee on Animal Experimentation provided approval (PEI/001/4087-4/2015).

### RNA extraction and reverse transcription

Total host + parasite RNA for all samples with exception of *Manayunkia* worms, was isolated using the Nucleospin RNA Kit (Macherey-Nagel) following manufacturer’s instructions. RNA from *Manayunkia* worms was isolated using guanidine/thiocyanate/phenol/chloroform extraction method^43^ to ensure higher concentrations of RNA compared to the column-based RNA extraction methods. A DNase digestion step ensuring elimination of genomic DNA was included into the protocol of the Nucleospin RNA Kit (manufacturer’s instructions). For *Manayunkia* samples, DNA was removed using the DNAFree Kit (Invitrogen). RNA concentration and purity were checked using a Nano Drop - 1000 Spectrophotometer (Thermo Fisher Scientific Inc.). All RNA samples with 260/280 ratio in range of 1.9–2.0, and 260/230 ratio in range of 2.0–2.4 were chosen for cDNA synthesis. Approximately 500 ng RNA was used as an input for synthesis of 20 µl of cDNA using the Transcriptor High Fidelity cDNA synthesis Kit (Roche) following the manufacturer’s protocol.

### Candidate reference gene selection and data mining

A list of eight commonly used cnidarian and other metazoan candidate reference genes were selected for this study: adenosylhomocysteinase (AHC1), beta actin (ACTB), eukaryotic translation elongation factor 2 (EF2), glyceraldehyde-3-phosphate dehydrogenase (GAPDH), hypoxanthine-guanine phosphoribosyltransferase 1 (HPRT1), DNA-directed RNA polymerase II (RPB2), 18S ribosomal RNA (18S), 28S ribosomal RNA (28S) (Table 2). Initially, this list included six more genes used as reference genes for cnidarians and other metazoans, such as eukaryotic translation factor 1 (EF1), NADH dehydrogenase iron-sulfur protein 2 ubiquinone (NADH), heat shock protein 70 (HSP70), ribosomal protein L11 (RPL11), TATA-Box Binding Protein Associated Factor 6 (TAF6), PHD finger protein 8 (PHF8). However, these genes were later excluded from the study/analysis, because either we were not able to find suitable homologues of these genes in our transcriptome/s, or primer design/ PCR was not successful. The eight candidate reference genes were mined from their respective parasite transcriptome data (RNA sequences) or from DNA sequences available in GenBank or at private databases. All available homologous amino acid sequences of these genes (GenBank) from common representatives of cnidarians such as *Acropora tenuis*, *Aurelia aurita*, *Hydra vulgaris*, *Hydra magnipapillata*, *Nematostella vectensis*, *Polypodium hydriforme*, and different myxozoan species (*C. shasta, Kudoa iwatai, M. cerebralis, Sphaerospora dicentrarchi,* *Thelohanellus kitauei, Buddenbrockia sp., etc.*) were combined for queries. The search was performed using the tBLASTn algorithm with the e-value cutoff set to 10^−10^. The top hits (highest e-value) were analyzed using the NCBI conserved domains platform (https://www.ncbi.nlm.nih.gov/Structure/cdd/wrpsb.cgi, to confirm their identity). To re-confirm the myxozoan origin of the mined sequences phylogenetic trees including other metazoan taxa (cnidarians, fish, etc.) were reconstructed using maximum likelihood methods in RAxML web-server (https://raxml-ng.vital-it.ch/#/). Details on chosen sequences are included in Suppl. Mat. 1.

**Table 2:** Details of selected candidate reference genes.

Gene Ids	Protein encoded	Generally accepted function	Accepted reference gene for	Used as RG for myxozoans
ACTB	Beta actin	Cytoskeletal structural protein	Commonly accepted reference gene^25,32,86^	*Myxobolus cerebralis*^32,33^
AHC1	Adenosylhomocysteinase	Homocystein synthesis protein	Corals^25^	
EF2	Eukaryotic Translation Elongation Factor 2	Nascent protein synthesis protein	Commonly accepted reference gene^67,68^	
GAPDH	Glyceraldehyde-3-Phosphate Dehydrogenase	Metabolic protein (glycolytic enzyme)	Commonly accepted reference gene^23^	
HPRT 1	Hypoxanthine-Guanine Phosphoribosyltransferase 1	Purine nucleotide synthesis protein	Commonly used for humans^22^	
RPB2	DNA-directed RNA polymerase II	RNA polymerase II transcription machinery protein	Humans^87^	
18S rRNA	18S ribosomal RNA gene SSU	Ribosome structural protein	Commonly used refrence gene^23,88^^,^	*Myxobolus cerebralis*^31^
28S rRNA	28S ribosomal RNA gene LSU	Ribosome structural protein	Commonly used reference gene^89^	

### Primer design and specificity of PCR

Gene-specific primers were designed to amplify short 70–150 bp regions suitable for RT-qPCR assays (Table 3). Primer pairs were designed with optimal Tm at 58–60 °C and GC content between 45–50%, using the NCBI online primer-design tool (https://www.ncbi.nlm.nih.gov/tools/primer-blast/). All primers were tested for specificity using conventional PCR prior to performing RT-qPCR. Details on PCR conditions are described in Suppl. Mat. 5. Primer specificity was determined by obtaining single amplicons of the expected size from infected samples and no amplification in uninfected fish and worm samples (indicating that primers are not annealing with fish cDNA). Controls without reverse transcriptase (–RT) were tested for genomic DNA contamination. The presence of infection was confirmed, and parasites identified microscopically and by specific published PCR assays^31,44,45^. The identity of PCR products was confirmed by sequence comparison. RT-qPCR primer specificity was also checked by running melting curve analysis (see Suppl. Mat. 3).

**Table 3:** Genes and their primer sequences used in this study.

Organism	Gene	Primer sequence (5′-3′)	Amplicon length (bp)	Melting T°(C)	GenBank access. numbers*
*Sphaerospora molnari*	ACTB	F: AATCCACGAGACCACCTTCG	149	59.75	see Suppl. Mat. 1
		R: CAGCAGCCAAACCGGTGATA		60.68	
	AHC1	F: TTCCCCATGGTGTCGAGAAA	138	58.94	see Suppl. Mat. 1
		R: TCAATGACACCTCGAACACAGT		59.9	
	EF2	F: TCCGGCAGGCAAGAAGGTTT	140	62	see Suppl. Mat. 1
		R: CCAAGTTGGATACGGATTACGAGT		60.44	
	GAPDH	F: TATCGACCTGGCCGTTACTG	118	59.63	see Suppl. Mat. 1
		R: GTTGCTGCTGTCAATGACCC		59.9	
	HPRT 1	F: TCTCATCTGTGACCGTGCTC	84	59.47	see Suppl. Mat. 1
		R: ACGCACAAAAACTCGGATCTG		59.47	
	RPB2	F: ATTAGTTACGGTGCCGGAGG	143	59.54	see Suppl. Mat. 1
		R: GCTGTGACATGGAAGATGCG		59.62	
	18S rRNA	F: ATCCCAGGTCGTATCCGCTA	73	59.89	see Suppl. Mat. 1
		R: ACTGCCCTGTTGATGCGATT		60.32	
	28S rRNA	F: ATCTGCTCGCACCTCATACG	143	59.97	see Suppl. Mat. 1
		R: CCGAGTTTGCTTGCGTTACC		60.11	
*Myxobolus cerebralis*	ACTB	F: TTGCCTGATGGTCAGGTGAT	110	59.01	AY156508.2
		R: AGTGTCTCGTGAAGTCCACTG		59.66	
	AHC1	F: GTTCAGCGTCGCTAAGAGGA	124	59.83	GBKL01003454.1
		R: GCCCGAGAGACACAGTCATC		60.18	
	EF2	F: ATGGATCCGGGCCTAACCTT	149	60.7	GBKL01021688.1
		R: CAAGTCCAGACGAACACCCC		60.6	
	GAPDH	F: GTGGCAAAACCCGCAACTAA	95	59.61	GBKL01017634.1
		R: TGTGCGTCGACAAACTGGAT		60.25	
	HPRT 1	F: TGGTGCTCCTGGTGAAGAAA	119	59.16	GBKL01050483.1
		R: GAGGTCGTCCATCCCAGTTT		59.39	
	RPB2	F: AATGGAGGGCTGGCTAAACG	127	60.39	GBKL01027608.1
		R: TAATCCGATGTCAGGGCACC		59.53	
	18S rRNA	F: TAGAGTGTGCCGAACGAGTC	85	59.48	EF370479.1
		R: GGTCCCAAGGCATCATGACA		60.03	
	28S rRNA	F: AGTCGAAGTAGAGCAGCGTG	141	59.83	AY302740.1
		R: CATCCTCAGGGATGCACTGT		59.45	
*Ceratonova shasta*	ACTB	F: GTCGGCAATTCCTGGGTACA	149	60.04	see Suppl. Mat. 1
		R: TCCAACCGGCATTTTTAGGA		57.41	
	AHC1	F: TTCGGTTACCACGACTCGGC	82	62.47	see Suppl. Mat. 1
		R: TGTAGTGGGTGGCTATGGTGA		60.55	
	EF2	F: CTGGATTCCAATGGGCAACT	147	58.14	KM392431.1
		R: AAATAACTCTTCGAGCAGTAGGT		57.34	
	GAPDH	F: TGGGGCTAAACAGTTGGTGG	152	60.18	see Suppl. Mat. 1
		R: GTGGACATTTGAAAGGAGGCG		59.8	
	RPB2	F: TGGAGGTTGAAGGTACGTGT	156	58.58	see Suppl. Mat. 1
		R: TCTGCCCCTTTATAGGACGA		57.54	
	18S rRNA	F: CCAAGTTGGTCTCTCCGTGA	121	59.32	AF001579.1
		R: CAAATTAAGCCGCAGGCTCC		59.9	
	28S rRNA	F: ACGTGAAACCGTTAACATGGA	132	58.16	FJ981818.1
		R: CCACTGGCCTTGAAGATTGT		58.08	

*Accession numbers are provided for the gene sequences that are available in GenBank/either mined from transcriptomic data under review, but see sequences in Suppl. Mat. 1.

### Quantitative real-time PCR

RT-qPCR was performed using the LightCycler® 480 Real-Time PCR System (Roche). Reactions of 25 µl were comprised of 12.5 µl of FastStart Universal SYBR Green PCR Master Mix (Roche, Germany, 2X conc.), 1 µl of each forward and reverse primer (10 µM conc., 0.4  µM final conc.), 5.5 µl of PCR grade water, and 5 µl of cDNA (generally at ~150–170 ng/µl dilutions). The cycling conditions were denaturation at 95 °C for 5 min, followed by 50 cycles of 95 °C for 10 s, 58 °C for 10 s and 72 °C for 10 s. Melting curve analysis was performed after each cycle to ensure primer specificity. All samples were amplified in technical triplicates and a mean value was calculated. Four (*M. cerebralis*) to five (*S. molnari*) biological replicates were used for each sample, with the exception of *C. shasta* (only 3 replicates available). qPCR efficiency was predicted for each gene based on the slope of a linear regression model^46^ using a series of 5-fold dilutions (1:5, 1:25, 1:125, 1:625). Standard curves were built using Roche Light Cycler 480 Software version 1.5.0 SP4. Generally, for best amplification results efficiency ranges of 90–110% and standard curve slopes of −3.58 to −3.10 were considered optimal^47^.

### Ranking and quantitative analysis of reference genes

**Figure 1:**
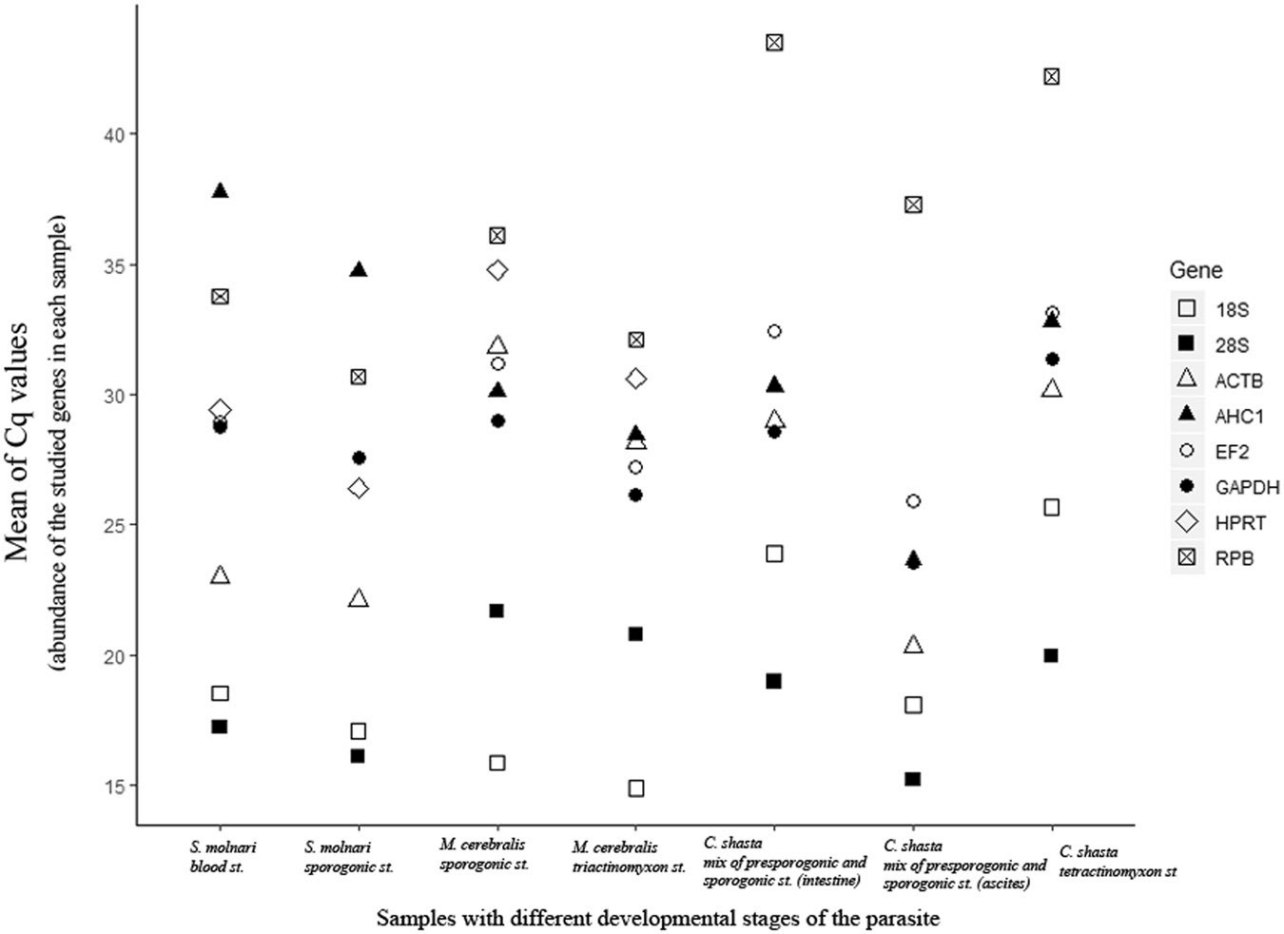
RNA transcription levels of candidate reference genes (in absolute Cq values) representing the abundance of the studied genes in each sample.

Differential expression levels and abundance of candidate reference genes within the sample was analyzed by a direct comparison of Cq (quantification cycle) values (Fig. 1, Suppl. Mat. 2). The stability of the candidate reference genes was analyzed using four algorithms: Δ*Cq*, NormFinder, geNorm, and BestKeeper. The comparative Δ*Cq* method manually compares relative expression of ‘pairs of genes’ within each sample. If the Δ*Cq* value between the two genes remains constant when analyzed in different samples it means that either both genes are expressed at relatively constant rates among those samples, or they are co-regulated (here we assume the stability of both genes)^48^.

NormFinder^49^ was performed using original Microsoft Excel-based software. It determines the stability of the candidate genes based on an estimate of inter- and intragroup variation. It calculates the most stably expressed candidate genes and suggests two of them as references.

**Figure 2:**
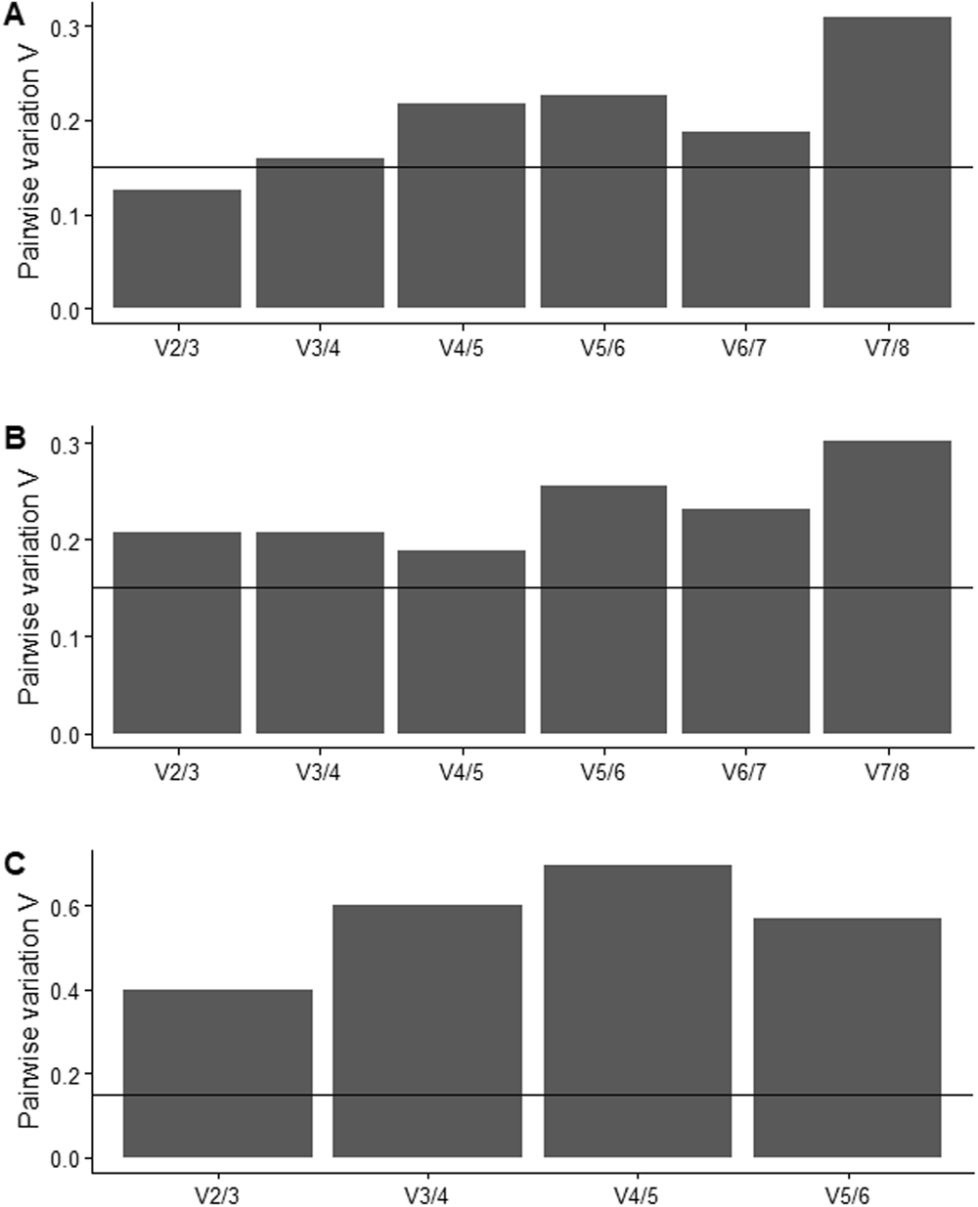
GeNorm pairwise variation (with threshold value = 0.15) suggesting optimal number of reference genes for normalization for A. *Sphaerospora molnari*, B. *Myxobolus cerebralis*, C. *Ceratonova shasta*.

geNorm was performed using the qbase + package software^50^. This program is based on the assumption that if the ratios between samples are uniformly expressed, non-normalized target genes should remain regular. The genes with the most irregular expression are excluded from further analysis while the last two remaining genes are selected as the most stable^51^. We used two values to interpret geNorm results: (1) geNorm M (geNorm expression stability value of reference genes, lowest M value indicates higher stability); and (2) geNorm V (pairwise variation). geNorm V further determines the optimal number of reference genes to be used in subsequent analyses. A Vn/n + 1 value is shown for every comparison between two consecutive numbers (n and n + 1) of candidate reference genes. As a general guideline (www.qbaseplus.com, qbase + manual, rev2017.04.27) it is stated that the benefit of using an extra (n + 1) reference gene is limited as soon as the Vn/n + 1 value drops below the 0.15 threshold, indicated with a horizontal line (Fig. 2).

BestKeeper was performed using the original Microsoft Excel-based formulas^52^. It calculates the standard deviation of the Cq value between the whole data set, and the gene with the lowest standard deviation (SD) is proposed as most suitable.

Finally, we used RefFinder (https://www.heartcure.com.au/reffinder/?type=reference accessed at 25 June 2019), a comprehensive software platform which integrates all four algorithms providing an overall ranking of the used genes.

### Transcriptomic data analyses

*De novo* transcriptome assemblies of *S. molnari* (unpublished) were used to observe expression of candidate reference genes in blood and sporogonic stages of parasite. We used *S. molnari* 11 samples (5 from blood stages and 6 from sporogonic stages) based transcriptomic data to estimate transcript expression values (TPM: Transcripts Per Million) using the Salmon software^53^. These TPM expression values were scaled and served to generate a cross-sample normalized TMM gene expression matrix (TMM: trimmed mean of M-values: scaling normalization that aims to account for differences in total cellular RNA across all samples), using the Trinity package^54,55^. We extrapolated TMM values for eight candidate genes expression values from the gene expression matrix and compared it across the 11 samples manually. Average values for each developmental stage were calculated. The most stable gene was considered the one for which the ratio between the average values of both developmental stages was closest to 1 (Table 4).

**Table 4:** TMM expression values of candidate HKGs for sporogonic and blood stages of *Sphaerospora molnari*. The genes for which ratio between sporogonic and blood stages are closer to 1 are considered the most uniformly expressed between two stages.

	Sporogonic stage					Sporogonic average	Blood stage						Blood average	Ratio between two stages
Biological replicate	biol repl1 (fish 1)		biol repl2 (fish 2)		biol rep 3 (fish 3)		biol repl1 (fish 1)		biol repl2 (fish 2)		biol rep 3 (blood stage mix of several fishes)			(Sp. average/ Bl. average)
Technical Replicate	tech repl 1	tech repl 2	tech repl 1	rech repl 2	no tech repl		tech repl 1	tech repl 2	tech repl 1	tech repl 2	tech repl 1	tech repl 2		
Sample name	3A	3B	4A	4B	5A		RNA1_L001	RNA1_L002	RNA2_L001	RNA2_L002	RNA3_L001	RNA3_L002		
GAPDH	81.29	73.27	62.23	77.49	47.42	68.34	29.83	29.25	83.18	84.50	106.20	118.88	75.31	0.91
EF2	269.91	230.57	193.22	282.79	143.82	224.06	74.14	73.91	331.30	331.03	313.90	298.76	237.17	0.94
18S rRNA	225.78	408.70	489.16	372.05	851.90	469.52	1301.10	1320.74	1154.50	1148.18	4751.65	4818.87	2415.84	0.19
HPRT1	1038.46	1134.00	1121.06	1068.91	1201.93	1112.87	73.16	70.86	371.42	371.66	438.28	470.23	299.27	3.72
RPB2	8.59	6.75	6.41	7.78	4.55	6.82	5.97	4.47	5.25	4.02	5.18	4.87	4.96	1.37
ACTB	3785.16	2868.36	2975.15	2916.40	3780.47	3265.11	4167.18	4235.62	10470.21	10430.10	10841.77	10873.28	8503.03	0.38
28S rRNA	273.61	504.40	392.36	390.14	1058.62	523.83	92.39	93.65	193.23	187.74	364.82	365.67	216.25	2.42
AHC1	22.11	23.63	18.93	13.89	40.43	23.80	1.93	1.84	5.80	3.98	0.29	1.39	2.54	9.37

## Results

### PCR specificity and primer efficiency

Primer specificity was confirmed by obtaining single amplicons of the expected size, together with negative results in uninfected fish and worm samples. Primer specificity was also confirmed based on the occurrence of a single peak in the melting curve (Suppl. Mat. 3). Absence of genomic DNA contamination was confirmed by no amplification in –RT samples. The efficiency of our candidate RG primers in the present study ranged from 88 to 129%, which slightly surpasses the acceptable optimum range (90–110%). However, we obtained similar efficiencies for the given genes in two different developmental stages of parasite.

### Cq data

Transcript abundance of each gene within each biological replicate was roughly estimated from the raw Cq values. The most abundantly expressed genes were 28S and 18S for all three studied species with Cq ranging about 14–25. The least expressed genes were AHC1 for *S. molnari* (Cq > 34) and RPB for *M. cerebralis* (Cq > 32) and *C. shasta* (Cq > 37.3). The rest of the genes fell in the range of Cq = 22–34 (Fig. 1 and Suppl. Mat. 2).

### Stability of candidate genes

**Figure 3:**
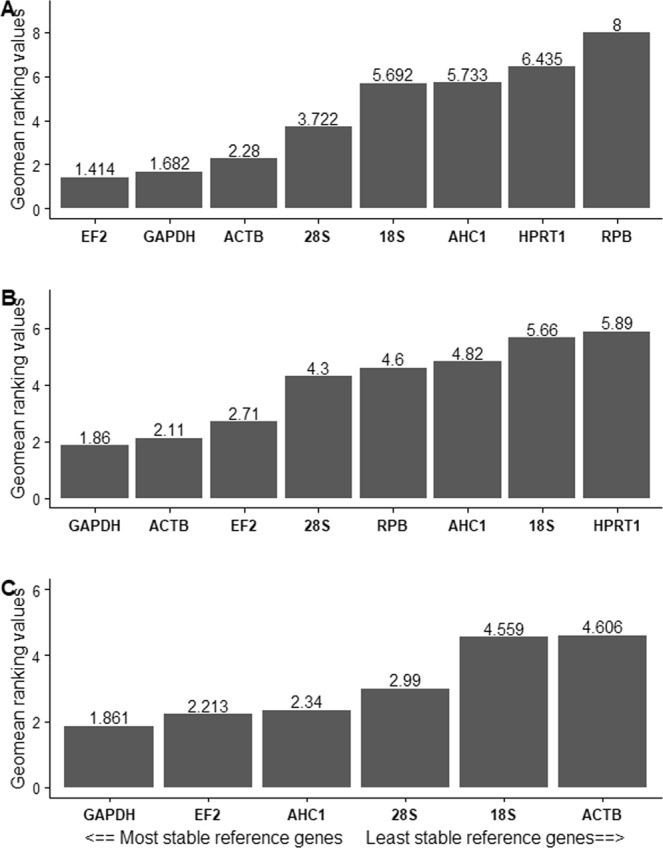
Stability of candidate reference genes expression for studied myxozoan species based on RefFinder comprehensive ranking: (**A**). *Sphaerospora molnari*, (**B**). *Myxobolus cerebralis*, (**C**). *Ceratonova shasta*. Y axis represents Genes Geomean of ranking values (lower value indicates higher stability). In X axis genes are ordered from high to low expression stability.

For *S. molnari*, the expression of EF2 and GAPDH was shown to be the most stable among the eight studied genes according to Δ*Cq* method (lowest average SD values 1.08 for EF2 and 1.12 for GAPDH), NormFinder (with lowest stability value = 0.18 for EF2 and 0.38 for GAPDH) and geNorm (lowest M = 0.37 for both genes). We obtained pairwise variation (geNorm V-value) V2/3 < 0.15, indicating that in this case 2 genes are sufficient for normalization, and that the additional inclusion of more reference genes will not provide a significant improvement for the normalization of target genes (Fig. 2A). While GAPDH was ranked in the second most stable place (SD ± Cq = 2.15), EF2 was ranked in the third most stable place (SD ± Cq = 2) by BestKeeper (Table 5, Suppl. Mat. 4). The same genes occur in the first 4 positions across all the algorithms: geNorm: EF2 > GAPDH > ACTB > 28S, Δ*Cq* method: EF2 > GAPDH > ACTB > 28S, NormFinder: EF2 > GAPDH > ACTB > 28S, BestKeeper: ACTB > GAPDH > 28S > EF2. To obtain a comprehensive ranking and summary of all the algorithms used, we loaded our raw data to the RefFinder web-based platform, which includes four above mentioned algorithms (Fig. 3). Overall EF2, GAPDH, and ACTB were observed as the most stable genes by RefFinder (Fig. 3A).

**Table 5:** Comprehensive ranking of studied genes using a combination of four algorithms. TMM ranking is based on normalized transcript expression values from NGS data. Lower ranking values indicate higher gene stability. Additionally, gene stability values generated by each algorithm are given in Suppl. Mat. 4.

Genes	GeNorm	NormFinder	BestKeeper	Δ *Ct*	Comprehensive ranking	TMM
***Sphaerospora molnari***						
ACTB	2	4	2	4	3	4
AHC1	5	7	7	6	6	8
EF2	1	1	5	1	1	1
GAPDH	1	2	3	2	2	2
HPRT1	4	5	6	5	7	7
RPB2	7	8	8	8	8	3
18S rRNA	6	6	4	7	5	5
28S rRNA	3	3	1	3	4	6
***Myxobolus cerebralis***						
ACTB	1	2	5	2	2	
AHC1	5	5	3	6	6	
EF2	1	3	6	3	3	
GAPDH	2	1	4	1	1	
HPRT1	4	6	8	5	8	
RPB2	3	4	7	4	5	
18S rRNA	7	8	2	8	7	
28S rRNA	6	7	1	7	4	
***Ceratonova shasta***						
ACTB	1	4	5	4	6	
AHC1	2	3	4	3	3	
EF2	4	1	3	2	2	
GAPDH	3	2	2	1	1	
HPRT1	Excluded from analysis					
RPB2	Excluded from analysis					
18S rRNA	8	6	6	6	5	
28S rRNA	7	4	1	5	4

We observed a similar pattern for *M. cerebralis*. geNorm suggested ACTB, EF2 and GAPDH with the lowest M value (M = 0.41, 0.41 and 0.57, respectively). Ge Norm pairwise variation showed middle stability, suggesting to consider 4 genes for optimal normalization (Fig. 2B). Δ*Cq* method (with lowest average SD values 1.11 for GAPDH, 1.12 for ACTB and 1.17 for EF2) and NormFinder (with lowest stability value = 0.27 for GAPDH, 0.56 for ACTB and 0.65 for EF2) also suggested the same genes. However, Bestkeeper’s ranking was different as GAPDH occurred in the fourth place (SD ± Cq = 1.69), ACTB in the fifth place (SD ± Cq = 1.95) and EF2 in the sixth place (SD ± Cq = 2). Overall, out of the eight *M. cerebralis* genes studied, the following genes ranked in the first 4 positions: geNorm: ACTB > EF2 > GAPDH > RPB2, Δ*Cq* method: GAPDH > ACTB > EF2 > RPB2, NormFinder: GAPDH > ACTB > EF2 > RPB2, BestKeeper: 28S > 18S > AHC1 > GAPDH. RefFinder suggested GAPDH, EF2 and ACTB as the most stable reference genes in comprehensive ranking (Fig. 3B).

For *C. shasta* the combination of the genes used in the analysis was slightly different. We obtained no expression or very low expression for HPRT1 gene in all samples, and very low expression (*ΔCq* > 40) of RPB gene in worm samples and thus these two genes were excluded from the final gene stability analysis. geNorm showed that AHC1 and ACTB and GAPDH had the lowest M value (M = 1.27, 1.27 and 1.54 respectively). However, pairwise variation (geNormV > 0.15) could not determine the optimal number of genes to be used for normalization. (Fig. 2C). Δ*Cq* method (with lowest average SD values 2.63 for GAPDH, 2.79 for EF2 and 2.86 for AHC1) and NormFinder (with lowest stability value = 0.94 for EF2, 1.15 for GAPDH and 1.92 AHC1) suggested EF2 and GAPDH, however Bestkeeper’s ranking was different as 28S occurred in the second place (SD ± Cq = 1.97), while GAPDH and EF2 were in fourth place (SD ± Cq = 2.87), and fifth place (3.06). Overall, out of the studied *C. shasta* genes the following genes ranked in the first 4 positions: geNorm: AHC1 > ACTB > GAPDH > EF2, Δ*Cq*: GADH > EF2 > AHC1 > ACTB, NormFinder: EF2 > GAPDH > AHC1 > ACTB, BestKeeper: 28S > GAPDH, EF2 > AHC1 (Table 5). RefFinder suggested EF2, GAPDH and AHC1 as the most stable reference genes in comprehensive ranking (Fig. 3C).

### Differential gene expression from transcriptome data

We have obtained TMM values for candidate reference genes in two *S. molnari* developmental stages (pre-sporogonic blood stage and sporogonic stage). We have calculated the closest to 1 value for EF2 (ratio between two stages = 0.94) and GAPDH (ratio between two stages = 0.91) indicating these two genes as the most stable of the eight studied genes (see Tables 4 and 5).

## Discussion

Expectations for real-time RT-qPCR are high as it serves as a first step of generating a data which will be a reference for the next steps of research applications (i.e. studies involving gene expression data).

While, numerous studies are based on qPCR data, in the past, only few reference gene validation studies were conducted. Many papers would use only a single gene as a reference, without verification of its utility under the used experimental conditions. This was especially common for majority of the articles concerning the analysis of RNA transcripts published in high impact journals in late 1990s and early 2000s in different organisms including myxozoans^23,24,31–33^.

In order to obtain reliable results, reference genes normalization and its rational interpretation are essential. It is complicated to determine if a two-fold variation in gene expression is of biological importance because this genetic variation can be triggered by intrinsic noise of biochemical reactions. Discrepancies with regard to organism strains, experimental design, and algorithms calculating differential expression further add to this noise.

While not everything can be controlled for, the first step for producing meaningful (true) data is careful evaluation of reference genes.

Since previous data evaluating the stability of reference genes in myxozoans are missing, in this study, we evaluated eight candidate genes for their suitability as a reference for future RT-qPCR assays in gene expression studies of myxozoans. We designed a comprehensive setup for testing these genes in a comparative approach by using RNA extractions from different developmental stages of the parasites’ dixenous life cycle, using three species from different phylogenetic lineages, covering the fields of biological and technical replicates and different calculation algorithms and methods by using RT-qPCR and transcriptomic data. Here, we discuss the parameters we used to ensure the best choice of reference genes, possible pitfalls that should be taken into consideration before final conclusions, and we provide recommendations for future RT-qPCR studies in this unique group of highly derived cnidarian parasites.

### Stability of the candidate reference gene and choice of algorithm

We used four well-accepted algorithms, geNorm, ΔCq, NormFinder, and BestKeeper in combination with TMM expression values mined in transcriptome expression data to evaluate the stability of the examined genes. Since these algorithms have different calculating approach, it might expect that the rankings of candidate genes could be different depending on the software applied. In previous studies we observed that the results produced by BestKeeper can oppose those of geNorm and NormFinder^56,57^.

Each approach has its strengths and weaknesses and there is no commonly accepted opinion on which one is the best. A consensus ranking of RGs is useful as it combines the data obtained from different algorithms and creates a meaningful outcome reflecting an overall agreement^58,59^. We used RefFinder for consensus ranking. We additionally checked the expression levels of examined genes in 6 transcriptomic datasets of highly proliferative and motile feeding stages *vs* 5 localized, predominantly intracellular spore-forming stages, for further confirmation of qPCR data obtained. In principle, RNAseq data can be used to identify good reference genes^60,61^ without previous selection according to published data. This is offering attractive perspectives regarding new RG discoveries, since the proposed workflows can be used for already generated transcriptomic datasets, regardless of sequencing technology, library size or organism^60^. With regard to myxozoans, there are not enough transcriptomic datasets of different developmental stages available for a single species and we hence compared preselected genes in new transcriptomic data of two different developmental stages of *S. molnari*. While other studies used TPM (Transcripts Per Million) or FPKM (Fragments Per Kilobase of transcript per Million mapped reads) values from RNA-Seq studies to analyse gene expression stability via CV (coefficient of variation) and fold change calculation methods^62–64^ we used a relatively straightforward approach, simply comparing already cross-sample normalized TMM values of the gene between studied conditions.

### Stability of the candidate reference genes and experimental conditions

Numerous studies showed that the stability of proposed HKGs vary across organisms and most importantly experimental conditions (i.e. developmental stages, drug/dietary treatment, temperature such as heat shock, cold stress, drought stress, etc.)^23,64,65^. Only genes that have stable expression under a condition to be analysed can be used as an RG for the given study. In the present case, we were mainly focused on providing RGs that are stable during myxosporean development, rather than e.g. under different temperature or dietary regimes. This is of particular importance to be able to investigate the stage-specific expression profile of parasite genes.

In our study, comprehensive ranking together with transcriptomic TMM calculations suggested that EF2 and GAPDH are the most stable genes across all the studied myxozoan species (Table 5). EF2 promotes the GTP-dependent translocation of the ribosome^66^. It is an essential factor for protein synthesis and thus, like other ribosomal genes, is assumed to have a constant expression throughout different tissues, different treatments or developmental stages of the organism. It has been shown to be the most stable gene for mouse DNBS disease treated and non-treated colon tissue^67^, or for plant tissues exposed to biotic and abiotic stress^68^.

GAPDH is one of the most used RGs in different organisms, including corals, fish, human, etc^23,29,69^. Although several studies showed that GAPDH can be regulated under some experimental conditions, i.e. gene expression in thermal and light studies^70,71^ it is reported to be a suitable reference for normalizing gene expression in various life stages, for instance in red algae^72^, plants^73^ or during the metamorphosis of free living cnidarian representatives such as corals (*Porites astreoides*^29^), which coincides well with the general scheme of our study. Our results suggested GAPDH as the first most stable gene for *M. cerebralis* and *C. shasta*, and the second most stable gene for *S. molnari*.

Actin is one of the most conserved proteins in eukaryotes, whose structure has been conserved despite the numerous actin isoforms reported with different biological functions^64,65^. Beta actin is one of the most common reference genes used in gene expression studies as it is known to be a key component involved in the development of cytoskeletal filaments^66^. It was listed as one of the best-performing reference genes in cnidarian/dinoflagellate studies^26^. Similarly, it was the best performing reference gene, along with 28S rRNA, in the parasitic apicomplexan *Theileria parva* from different host tissues^67^. Beta actin was used as a reference gene for *M. cerebralis*^2^ and a number of gene expression studies in free-living cnidarians^26–28^, however, it has been shown to be regulated under various experimental conditions and was redefined as an unsuitable reference gene in some cases^23^. Multiple actin isoforms are known from myxozoans^74,75^ and in *S. molnari*, the expression level of two highly divergent isoforms differs about 15-fold, since likely only isoform 1 is responsible for the unique parasite motility during proliferation in the blood^75^. These highly variable expression levels of different isoforms suggest different functions and even though we used actin isoform 2, which was expressed at a low level, for the design of our qPCR assay, our results did not support beta actin to be an optimal reference gene for *S. molnari* or the other myxozoan species studied. It was ranked only in third place for *S. molnari* and *M. cerebralis*, and in sixth for *C. shasta*. TMM data also placed it in the fourth place for *S. molnari*. Actin isoforms are very similar to beta actin and gamma actin, differing only by four biochemically similar residues and being conserved from birds to mammals^76^, however, actins show highly divergent DNA sequences in myxozoans^75^. Hence, the possibility of misidentification of the same actin isoform in different species of the highly derived myxozoans gives an additional reason for excluding beta actin from qPCR analyses in myxozoans.

Alongside with beta actin, ribosomal genes especially 18S and 28S rRNA are traditionally used as reference genes. Being structural components of small and large eukaryotic ribosomal subunits (40S and 60S), they are one of the most basic components of eukaryotic cells. However, the suitability of 18S and 28S rRNA as a reference gene varies in the literature. In myxozoan studies 18S was used for phylogenetic studies and for detection and quantification of parasites in environment or host tissue^34,35^. Thus, we have included it in our study to test its utility as reference gene, since it is shown to have high stability. However, despite its high stability in a number of organisms^77–79^ many studies indicated that these are highly expressed genes and are often unsuitable for comparison. It can be challenging to compare them with target genes expressed at a low level which can lead to erroneous results^23,80^. Indeed, 18S and 28S rRNA were the most highly expressed genes (Cq < 21) in our study, except for *C. shasta* (Cq for 18S = 23.9–25.6 in intestine and worm samples, respectively). In our study 28S was the fourth most stable gene for *S. molnari* and *C. shasta*.

### Stability of the candidate reference genes and the influence of used methodology

Despite RT-qPCR being one of the most reliable techniques to accurately measure the expression level of a gene, there are number of factors that may affect the consistency of expression data. For instance, different dyes can influence PCR inhibition in a concentration-dependent manner and can have effects on DNA melting temperature or can preferentially bind to certain DNA sequences^81^. PCR efficiency can be influenced by PCR inhibitors present in the sample or by non-optimal primer design. This information is critical, since these factors can produce different results even if the experimental design or study organism is similar (see also notes in Suppl. Mat. 5). Overall, we obtained consistent results for the suitability of RGs in three myxosporean species from different phylogenetic clades: EF2, GAPDH together with ACTB were the most stable genes for *S. molnari* and *M. cerebralis*, and EF2, GAPDH and AHC1 for *C. shasta*. Additionally, comparable TMM values of *S. molnari* demonstrate the robustness of our predictions. Thus, it can be hypothezised that these genes will also have stable expression in other related myxozoan species. These results are useful in particular for studies involving developmental stages of these parasites, however furthermore it would be of great interest to check the stability of these genes under different experimental conditions such as temperature, drug treatment, different dietary treatment, water quality, etc.

### Possible pitfalls of detecting less abundant transcripts and further recommendations

Likewise, highly expressed genes, such as 18S and 28S rRNA, reference gene may not be suitable to use in gene expression studies, if the gene of interest (i.e. target gene) has low expression in comparison to the reference gene. For instance, in our study, suggested reference genes (EF2, GAPDH) showed a suitable expression range (Cq = 23–33) in all samples (see Fig. 1, and Suppl. Mat. 2 for the abundance of each gene in each sample). However, we noticed that all the investigated genes in these samples showed low expression levels, which could be related to the low amount of parasite concentration in the sample or PCR inhibition, which may occur in guts and soil samples^82–84^.

PCR inhibition is something to be aware of, especially in invertebrate host extractions procedures, and need to be carefully evaluated to ensure sufficient representation of quantifiable transcripts in subsequent myxozoan studies.

Several attempts (i.e. using inhibitor removal column and reagents, see details in Suppl. Mat. 5) were undertaken to reduce inhibitory effect in this study, which did not improve our overall results. Another useful way to evaluate the inhibitory effect is to use serial dilutions, since inhibitory effect can be lost in high dilutions. However, for the samples where the parasite concentration is already extremely low, dilution may not always be an optimal solution.

While we cannot exclude PCR inhibition as the reason for low detection of some of our genes, it is possible that the high Cq values observed are simply related to low parasite concentrations in the sample or these transcripts are expressed in low levels.

In either way, detecting less abundant transcripts remain an open question, especially in invertebrate samples, and more analyses (i.e. using new inhibitory effects removal kits, testing different concentration of RNA, carefully evaluating the primer design) may help to clarify this problem.

Finally, using a single reference gene for gene normalization is generally less reliable than the use of a set of genes^85^, and based on our data, we propose using a combination of at least 2 to 3 genes for myxozoans. To our knowledge, this is the first study to validate RGs for myxozoan species, and we are convinced that the results presented here serve as an essential aid for subsequent gene expression studies of this group of extremely derived parasites.

## Supplementary information

Dataset 1, Dataset 2, Dataset 3, Dataset 4, Dataset 5

## Data Availability

The data that support the findings of this study are available in Supplementary Material.
