# Author Correction: Selection of suitable reference genes for gene expression studies in myxosporean (Myxozoa, Cnidaria) parasites

**DOI:** 10.1038/s41598-019-54998-y

**Published:** 2019-12-04

**Authors:** Anush Kosakyan, Gema Alama-Bermejo, Pavla Bartošová-Sojková, Ana Born-Torrijos, Radek Šíma, Anna Nenarokova, Edit Eszterbauer, Jerri Bartholomew, Astrid S. Holzer

**Affiliations:** 1 Institute of Parasitology, Biology Centre, Czech Academy of Sciences, 37005 Ceske Budejovice, Czech Republic; 2 Department of Microbiology, Oregon State University, Corvallis, Oregon, USA; 3 Centro de Investigación Aplicada y Transferencia Tecnológica en Recursos Marinos Almirante Storni (CIMAS), CCT CONICET – CENPAT, San Antonio Oeste, Argentina; 4 Faculty of Science, University of South Bohemia, Ceske Budejovice, Czech Republic; 5 Institute for Veterinary Medical Research, Centre for Agricultural Research, Hungarian Academy of Sciences, Budapest, Hungary

Correction to: *Scientific Reports* 10.1038/s41598-019-51479-0, published online 21 October 2019

The original version of this Article omitted an affiliation for Anna Nenarokova. The correct affiliations for Anna Nenarokova are listed below:

Institute of Parasitology, Biology Centre, Czech Academy of Sciences, 37005 Ceske Budejovice, Czech Republic.

Faculty of Science, University of South Bohemia, Ceske Budejovice, Czech Republic.

Additionally, this Article contained typographical errors in the Acknowledgements section.

“We acknowledge financial support by the Czech Science Foundation (grants numbers 505/12/G112 and 14–28784 P), the European Commission (project reference 634429, ParaFishControl), Ministry of Education, Youth and Sports of the Czech Republic (project no. LTAUSA17201), the Hungarian National Research, Development and Innovation Office (project no. NN124220) and Consellería de Educación, Investigación, Cultura y Deporte, Valencia, Spain (APOSTD/2013/087). We thank Stephen D. Atkinson and Julie Alexander (OSU, USA) for the help in *Manayunkia* worm sampling.”

now reads:

“We acknowledge financial support by the Czech Science Foundation (grant numbers 19-28399X (to AK) and 14–28784 P (to GAB)), the European Commission (project reference 634429, ParaFishControl (to ASH)), Ministry of Education, Youth and Sports of the Czech Republic (project no. LTAUSA17201 (to PBS)), the Hungarian National Research, Development and Innovation Office (project no. NN124220 (to EE)) and Consellería de Educación, Investigación, Cultura y Deporte, Valencia, Spain (APOSTD/2013/087 (to GAB)). We thank Stephen D. Atkinson and Julie Alexander (OSU, USA) for the help in *Manayunkia* worm sampling.”

These errors have now been corrected in the HTML and PDF versions of this Article, and in the accompanying Supplemental Material.

