# Supplementary material for: Selection of suitable reference genes for gene expression studies in myxosporean (Myxozoa, Cnidaria) parasites
Source: Sci Rep. 2019 Oct 21;9:15073. doi: 10.1038/s41598-019-51479-0 (PMC6803631; doi:10.1038/s41598-019-51479-0)
Supplement: Supplementary file 1 — Dataset 1, Dataset 2, Dataset 3, Dataset 4, Dataset 5 [file 41598_2019_51479_MOESM1_ESM.docx]

**Supplementary material**

**Selection of suitable reference genes for gene expression studies in myxosporean (Myxozoa, Cnidaria) parasites**

Anush Kosakyan^1*^, Gema Alama-Bermejo^1-3^, Pavla Bartošová-Sojková^1^, Ana Born-Torrijos^1^, Radek Šíma^1^, Anna Nenarokova^1,4^, Edit Eszterbauer^5^, Jerri Bartholomew^2^ and Astrid S. Holzer^1^.

^1^Institute of Parasitology, Biology Centre, Czech Academy of Sciences, 37005 Ceske Budejovice, Czech Republic.

^2^Department of Microbiology, Oregon State University, Corvallis, Oregon, USA.

^3^ Centro de Investigación Aplicada y Transferencia Tecnológica en Recursos Marinos Almirante Storni (CIMAS), CCT CONICET – CENPAT, San Antonio Oeste, Argentina.

^4^Faculty of Science, University of South Bohemia, Ceske Budejovice, Czech Republic.

^5^Institute for Veterinary Medical Research, Centre for Agricultural Research, Hungarian Academy of Sciences, Budapest, Hungary.

*anna.kosakyan@gmail.com

**Supplementary material 1. Sequences of candidate reference genes mined from transcriptome data (*S. molnari* and *C. shasta* transcriptome NCBI SRA Acc. NumberSRR6782113, *M. cerebralis* transcriptome from GenBank with accession number GBKL00000000.1 is used) or retrieved from GenBank for designing gene primers.**

***Sphaerospora molnari***

**ACTB** (actin2 sequence from Hartigan et al. 2016)

>TRINITY_DN52484_c0_g1_i1

CGCTCTTCCTCACGCCATCCTCAGATTGGATTTGGCCGGTAGAGATCTCACTGAATACCT

CATGAAGTTGCTCACCGAACGTGGAAACACCTTCACCACCTCAGCCGAAAAAGAAATCGC

CAGAGATATCAAAGAGAAACTCTGCTACGTTGCTTTGGACTTTGAACAAGAAATGGCTAG

CGCCTCCTCCTCCAGCTCATTGGAAAAACCCTATGAGATGCCCGACGGACAGGTTATCAC

CATCGGTAGTGAACGTTTCAGATGCCCAGAAGCTCTCTTCAAGCCCAGCCTTTTGGGTAA

GGAAATCAAAGGAATCCACGAGACCACCTTCGATTCCATCTCTGCTTGCGATGTCGATAT

CAGAAAAGATCTGTACGCCAACACCGTCCTTTCTGGTGGTACTACTATGTACATAGGTAT

TGCCGACAGAATGACCAAAGATATCACCGGTTTGGCTGCTGCTACCATGAAGATTAAAGT

TATTGCCCCACCCGAGAGAAAATACTCCGTCTGGATTGGAGGTTCCATCCTTGCTTCCCT

ATCCACCTTCAACAGCATGTGGATCACCAAACAAGAATACGACGAGTCAGGTCCTTCCAT

TGTTCATCGCAAGTGCTTCTAAATTATATTAGATCGTATCACATATGCCATCAGATTAAT

TTGCTTGACTTGTGCGAAAAAAAAAAAAAA

**AHC1** mined from transcriptome

>TRINITY_DN754_c0_g1_i1 len=1610 path=[3151:0-1162 3156:1163-1232 3150:1233-1609] [-1, 3151, 3156, 3150, -2]

AATTAATCCCACTCAACGGATCTCAGCCTGAAAAACTCTTCTTTTTATGAGTCGTCTTAG

GCAGAAATCATTATCTCCAGACGATATCAAAAAAGACGGAATTTCCCCTCGAATGGAATA

TCTAAACTTATCCATTCCGCAAGATTACTGTGTGAAGGACATGTCATTGGTCAAAATTGG

AAAAGCCGAAGTTGAATCTGCAACTGATGATAATAATATGCTGGCGATAAAATTGTTGAA

AGAACAGATTCTGGATTCATTTTTAAAAGGAATTGTGATTTGTGGCTGCACTCAAATCAC

TTCATCGACTGCTGTTTTGATCGAAGCTTTGAAACATTTTGGAGCCACAGTTCGATGGTG

TAGTCCACTCTTCAGTCCAGTTGATGACAGTGTCGCTGCTTTTCTTGCATCTTCAGGAGT

ATCTGTGTATGCCTGGGAAAATTCTGACGAAACTGAAGATTCCCCATGGTGTCGAGAAAA

ATGTTTGGACGTCCCAGGAAATTCCCCGATCATTCTTCTTGAAGACGGAGGTCATCTCAG

TTCATTTCTTTTGTTAAATTCCCATGAAAAATTACACTGTGTTCGAGGTGTCATTGAAGA

TAGTTTAAATGGCATTCTAAGACTTGCTCAGTTCCATCACAAATGCCAACTTTCATTTCC

TGTGATAAATACGTCTGAAGCGCTTATTAAGAAATTTTGCGCAACATTGTTTATTTCGAA

AGAATCAGTATTGGAATGTATTCGTCAATGTAATGATGTGATGATCAGTGGATCCAATTT

TTTAATCTGCGGTTACGGACATCGAGGAAAAATTTGTTGTGCCACTTTGAAACAACATGG

TGGAGTTGTGATGGTATCTGAAATCGATCCAATCTGCGGATACGAAGCAATAATGGATGG

TTATGAATTAGTGAATATTAATGATGTTATTTCCAAAGTCGACGTTATCATTACATGTAC

TGGAGGTATCGAAGTAATCAATCGATCAATCTTGGAGCGAGCAAAACATGGATGCATTGT

CATGAATCTAGGAAAATCTCATGTAGAAATCGATACGAAATCACTTCAGGCTCCTGACAT

AATCTGGGAAAGATCCGATCTCATTTCCATTAGCCATTTGGTATTCAAAAACAGCAACAA

ACGAATCACTTTATTGGCAGATGTATGTGATTTCTATCATTCACTGATATGAAAATTTTT

GATGAAACAGGGACGCATGGCCAGTCAGGAATTTTCCATTTGTCCAATGCGTCTGCAATC

CACATTTGTTTGCACCATGATTCTGGGAGTGATGGAAATTGTAACATCAAAACCTGGACG

TTATTCTCCGGGAATATTCATGTTTCCAAGAATTTTAGATGAACAAGTTGCTCAGTTGTT

TATCAAATCTTCCAGAGGTCATTTGACAACTCTAACCTCTGAACAGGCGAAATTGTATGG

AATTTCACCCAAAGGTCCTTTCAAAACTATACATTTTCATTATACCTCACCTTTGGTCAA

TTCTACTTCGGAGGATAACTCTTGAGGCAGAACTTTCCGTTGTGATTTTTAAATTTTACA

TCCTTAAACAATTGTGATATAATAATTATTATTATTATTATACCCTAAAA

**EF2-like** mined from transcriptome

>TRINITY_DN37176_c0_g1_i1 len=2825 path=[5605:0-2824] [-1, 5605, -2]

TTTTTTTTTTGATACCAGTTGAAAATATACGGCATAAATTTAAATAATAAATGGCGGCAA

ACAATCAAATTTATCAGACGATCTTAAAGACGAGAGTGTAACAAACTACAGAACACTCGT

TAAATCTCTACCATTGTTTGGTGAGGATTGGATCGTAATAGTCACTAGCAATTGGCAATT

TCTCATTCATCTTGCGACGCTTTCTGATCGCACGCACAATTGATGAAGTGTTGACCATGA

ATTCAGTTTCCACTGGAGGATCATAAGGATCTTCGTCCATCATAGCCCAATGAGAGAATG

AGCAGTTAGGGAAGGCTGTTCCACTGGTTGCTTTTCTCATGTGTGGCAAGAGATCGAATG

ATCTTCTGACAGGGATGTGAGCGATGGTGACCATAACTGAATTGGTTTTATCATAGGCAT

CAACGGGAATTCCATTACGTTTGCTAATAACCTCATTCACGTTGCTTTGTTGAGTTCTTG

GATGCTCAACTTCAACCTGGAAGAATGGTTCTACCATTCTGGCACCATTGATCAACAAAG

AAGCAGTGCACGCTCGCTTCGAACATTGGGAAATCATACCAGAGTTGCGATGAACGACAT

CGGAGTGAAAACTGGCATCCTCAAGATTGAAAATGACACCACGCATTTCAGAGCCAGTGA

GAGGTCCTTGGAAGCAAACTTCAGTGAATCCTTTGACGATACCATCTTTACAATCTTTCA

GATAGTCCACACCAGCTGTGGCATTGTTTATGAAGTTGCATCCGTAACGAGTAAGGTTGG

TTTGTTCTGGAGCAAATCGCCAGACATCTGAAGCAAATTGATCTGGAACTCCCGCTTGCA

CAATAGCCTTTGCTCGCTCTTTGGCAGTCATTTGAGTGTTGATGACTCCGTCTTCAATTG

CCTTGAGACAAGGCTCTGCCATGGAAATGGCTCTCATTCTCAGTCTGTTGTGACCGTTAG

AAGACTTGGTGACCACACAAATGTAATTGCTAGGTTCACTCTTCGTATCGATAGTTTCAC

GATATTCGACAATTGGTTCTCCAATGTCAATCTTGACACCGGGAGCGTACATTTCTTGAA

GATCCTTCAAGTTGATATCCAAATGCATTTCACCAGTTCCACTGATAACGTATTTACCAT

CTTCAATGGACTTATAACTGATTATGGGATCGGCTTTAACCATACTGTTCAAACCGTTAA

CCAATTTGGTGAGATCAGAACTGTTCTTAGGCCAGATGACTCTCTGCATAATTGCAGACA

CACTGAATTTCATCGTTCTAATAGGTTTGGCACCAGGTTCACTGCAGATGGTACCAGATC

TCACAAGCCATTCGTCAATACCCATGAGAGCGCAAACGTTTCCAGCAGGAACAGAATGAG

TCGGCAAGGTTTTGCTGGCCAAACAAATGCACACACGTTTAGCATTTTTGCCCTCATATG

ATTTGCTGCTACCCTTCATGTCAGAATCAGCTGGGATGATTGTGAATTTGCTGCTAGTAC

TGATTGTTCCAGAAAAGACACGGCACATAGCGAAAAATCTATTGCTTTCGGAAGATGGGA

CCATTTTCGACACGTAAATCATCAAAGGTCCGTTCGGGTCGCATTTTTTGATGGCTTCGG

CCGAGGGATCATCATGTTCACCAGTATACAAAAGTGCTTCTCTGTATGCCTGGGCTACCT

TTGGAGATGGAAGATGTTTGACGATGGATGAAAGAAGTGAGGTTCCGGCAGGCAAGAAGG

TTTTCATCACAGCACGAAAGATTTCATTCTTTGTTTTGCCAGCCAGGTCGCTTGGAACAG

TGTAGTCGTGTCTCAATCGTCTGATAATCTTTTGCATTGACTCGTAATCCGTATCCAACT

TGGAGACCTCTCGGATGATATCGAGAATGAAATAACAGGCCATTCTATCTCTGTTCTCTC

CAGGTTTTTTGACAACTTTCTTGGTCTCGTGGTCGAAATAGTATTCACCCCATAAGAATT

TTGAAAAGTCTTGGGAGGCCTTGATGAACTCCTCACTTCCTCGATCGATTGGCATGCCTC

GATCAGAGAATTTTCTGGCGGTCATCATGTCGGATACGTCCGAGATTCTGAAGGCCCAAC

AATTCAATCCTGAACCAAATACTACGCTGTCATTAAGTGGATCGACTCTATAGTTTCCTA

ATGGAGAATCTTCTTCAACGTTTGTGGCAATAATGACGTTGATGCTTTCGATATCTTTGT

TCATAGTTTGGTATACCTCTTCAGATGAAGCTCCTTGATTGAACGCGCTGTCCATCTTGT

TCATAAAAAGAATAGGTTTTGAAAGTTCAGATAGCGCCTGGACCAATACAGTTTTTGTTT

GAATAGAGACATTGGCCACAATAGAAACCACAACCAAAGCACCGTCAGTGACACGAAGAG

CTGCAGAAACTTCACTGGAAAAGTCAACGTGACCAGGCGAGTCAATAAGGTTGATGAGAA

ATTCGTTTCCATCACTCTTCACCTTCATTCGTTTCAGGTCCAGCTCGTCAACCTTGTAGT

ACATGGATATAGCGGTTGATTTGATGGTTATACATCTGTCCTGTTCGTCCGCTCTGGTAT

CAGTGAATCTTTGATTTCCAGCCTTGTGATCAGAAATAATACCGGCCAGTCCAACCAAAG

AGTCAGTAAGTGTTGTCTTTCCGTGGTCCACGTGAGCAATGATGGAGATATTTCTAATGT

TATCCTGTTTGTCCATCAGAGCTCTGATAGTAGTCTCGTCTAAGCTCGCCATTCTTTAGT

GGTCTTTAAAATTCAAGAGTCGCAACGTCAAGCACACACGAGAACAGCCGCACACGCTGA

GTTAG

**GAPDH** mined from transcriptome

>TRINITY_DN39930_c0_g1_i1

TTTTTTTTCAGCGCATAAAATTTAAAAATTACGATTTAAGCGAAATTGATGGAGCATACAACTATCAGATGAGACTCTATGGAAGGAATTAGATGCGACAGAATATTTATGCTTCTTGTTCCCTGGAGTGGAAATAGCATACCATGTCTAGCAGGCGATTGGCGTATCCATATTCATTATCATACCAGAAAGACAGTTTGTAGAATTTACGATTGAGGGCAATACAGGCCTTGGGATCGAACCAAGCAGATTTGGATGTTGTGTTGAAATCATTAGAAACGGCATTGTGCTTAACAATTCCGAAAAATCGACACTTTTCCTGTGATTTTGCAGCAGCAATAAACACTCTGTTGATGTCCTCCATGTAGCACTCCTTTTGAAGATTAACAAATAGATCTAGAACTGAAACATTCATAACTGGAATTCGAAGAGCGATTCCATTGATTTTTCCGGCGAGTTCGGGAAATACTCTAAGGATTGAGCAAATAGCGCCGGTGCTGTATGGAATGATGTTCTGAACGGCACTTCGACCACAACGTTCACCAATTTTGGATGGAACATCGAGAGGTTTTTGGGAATTTGTCATTGAATGGACCGTGGTTATGGATACAGACGCAATTTTAAAGTTGTCATTTAGAATTCTGCAAACAGGAGCAAGACAGTTGGTAGTGCATGAACCCATTGATACTACTTGCATGGTTTTAGGGTCATAATTCTCTTCATTCGCTCCGAGCACAATCAAAGGTGCGTCTTTGGAGTATGCGGAAATAATAACACGCTTCACGCCTTGCGTAAGGTGCTTGGATGCGGATGCCATCGTGGTTAATCTTCCAGAGCATTCTACAATGAAGTTGGCCTCATTTGTATCCCAGTGAATAGCACTTGGGTCGGTTTCTGTTAGATGTAGAAACACGATCATGCCTTTGTTGATATCGACCTGGCCGTTACTGTGTGACACAGGAACCCCAAGACGTCCATGGGTAGAATCGTACTGCAATAATTCTACCAGAGTGTCAGTTTGCATGTGAGGGTCATTGACAGCAGCAACCGTTAAGCTTTTTTCTCCTTTCAGATGTTTCTCAATCACAGCCCTCATCAAGGCTCTTCCG

**HPRT1** mined from transcriptome

>TRINITY_DN10840

GCTAAATAACCCTCTGAATTATATATATATATATATATATATTAAACATGTGACAAGTGT

CAACAAATAATCCGCGAAAATGGCTCACAAATCGGAGGGAAAAGAAATGATTTAGAAATC

TGGAAATCATTGAAAGGAGACTGAAAAATTCTATTCCAGACGAGGTAATATTATGGCAAA

ACACCACGGTTTTTAAAAACACGTGACACCTGGGCGGTTGCGACCGAATCCTTTTCGCTC

TCACACATCAATGATCGTGATTCCAGACGGTGCAGGATATGATCCAAAATTCTTTTGTGG

CCCAACTGTTGATTATGAAGACATCGAATGCATCTTAGTTCCACACGGTCTCATCTGTGA

CCGTGCTCGTAAAATGGCGTTGGACATTTGGAATGCCATGTCTTATGATCGCAGATCCGA

GTTTTTGTGCGTAATGAAGGGCGCCAGCAATTTCTTCCATCTTCTAATTTCCTATCTTTC

CGATATCAACGAATATGCTGCTAAGCCAAAGGTGTTTGACACAGACTTTGTTCGTACCTC

CAGTTATCATAATACAGATAAGAAAGCCAACTGTGAAGTCACAAACATTGATACTTTGAA

CCTGCAAGATAAGGATGTTTTTCTTGTTGAGGATATCGTGGATACAGGTGAGTCCATTCA

GGCTCTCTCTCAGAAAATCATGACCAAGAATCATCCTGCCAGTATTAAACTGGTTTCATT

GGTTCTTAAACGTGATACTACAAACACAACCGTCATTCCTGAATTTTCTGGATTTTCTGT

TCCAAAAAAGTGGTTGGTTGGATTTGGAATGGACCACACTGAGCGATTCAGACATTGGAA

TCACATTTGTGTGATGTCATCAACCGGAATCGCAAAATACAATCAATCATAACCTGCCGG

TCATCATCATCATCATCATCATCATCTTCGAATTCCAGTTTCTTTAAGTTTCAATA

**RPB2** mined from transcriptome

>TRINITY_DN9928_c0_g1_i1

TGATCAGAAAAAATGAAAATATTATACGAAACTATTTTACAAACTTGTAACAACAAGAATCCATTACCTGGAAGCTAGTTCAAATTTCAAGGAAATATTAACCGCGGCCAGTTCTGAAACGAGATATTTCAACACATACGGAATCATTATGCGCTTAACAGAAGCTCCACTCTTGCAAATGCTGCATATCAAAGATTTTTGTGACTGTGAATTTCTGGACTTGTTACTTTTACTTTCTTGAATACCATGAAGAATAGAAATTCGCTGGAGATGACATGAAGAAATATCACGGCATGTTGTACAAATAAGAACTTCACTTTTATCGGAACAATGGAAAAGACGATCTTGAAGAAGAAAAGAAGTACCATGAGCAATTAGAGCATCTCGTTCCATTTCACCAAATCGAACTCCACCGTGTCTCTTACGCCCATGAACAGGCTGATGTGTGAGTTGATCAACTGGTCCGGTGGAACGTACCTGGAATTTATCAGAGATCATATGTCTTAGCCGCTGATAATAAACAATTCCAAAAAATATTTCAGCTTGAAGTTGGAGGCCGTTGGTACCACAAAATAACATTTCAGTTCCATAATGATTAAATCCAGCTGATGTGAGTTCTTGGCCGAAGTGTTCAACAAAATTATGAGATATTTCTTGGAAAGGTGTTGAGTTTTGTACCTTTCCATAAACCGCTCCTGATTTGGCAGCCATAGTTTCGATGATCATCCCAATCGTCATACGGGATGGAAAACCGTGAGGATTGAAGATGATATCAGGAATAATTCCAGATTCGGTGAACGGCATGTCTTCAGAAGGCCAATTGGAACTCAACACACCTTTCTGACCATGACGACTTGCAAATTTATCTCCGACAATCGGATTTCGATTCATTCTTAAGCGAATATTAACGCGAAGTAGTTGAGTAGGATCTACTCGTCCCAATGCGGCTACATCTTCGACATACGCAATATCGTCACCCTTGTATTTACGAACAGTAAAAATGTTTGTACAAATGTCATAATAACAGTAGAATGGATCATCGTATTTCAAACGAGTTCCGATCATGGGAAGACCTTCCACATCCAGATGAGAGGTTAGAGCTGGTTTCTTAGGATCTCGTCCGAATCTGAGATTAGTTACGGTGCCGGAGGAGGAATTTCCACTGGAGCCTTCTTGAACCAAATCAATCAGTATTGATTTGTAAATGGACGCGTGAGCAAACCCACGTTCCAGGGCAGATTTATTGATAACCATCGCATCTTCCATGTCACAGCCTGTATAAGAAATGACAGCGACAACTGCATTGAATCCCTGGGGATAGTCATCCATATGGACAAGTGAAGTATTGGCGTTATTGCAAATGGGACTTTGAGGAGTCATCAATCTGTAGATTTTATTGTCGAACCGATGCTTGTATGAATACATCGATGTTCCGAGACTTTGTTTCGCCATCTGACATTGGTACATGTTTCTTGGACTCTGGTTAAGATCAGAAAAAGGAATTAAGGATGCGGCATCGCTAAGAATCAAAATTGGATCGATTTCCATGTGAGAATGTTCCGGTGTATCTTCTTTTTCCGATGCTGAAATATTCATAAAAACTTGCTCGTGGCATCCGATCATTTCAACGCACTTCAGTGATTTATTCCACACAGGTCGCACCGGTCGGCACGGCGATGTAAAAATATACAAGCCTGGAAATTCTGCACCACCACCATCTTCGTATTTTTCCACTAATGAGATTTCGTATTGTTCATTTTTCTGGATCTTGAATTCTCTGAGAATTGAAGTGATTCGAAGAGCATCTTCGTTTAGAATCATTCCAACCACGTTTCCATCAATCATCACATGGATGTAGGAATTGTCAAGAGATAGTGGCATCACTACCACTCCGTCACAGGATTGAAGACCCAGTGATTTAAGACAATCAATTATTTCATCGGTACTGACCTGTTGAGTGGATATTTCACATGTTAAAGCCAAATGATTTAATAAACCGCAAGGTGCCCCATCAGGTGTGTGCACAGGACAGATAAATCCCCAACATTCTGGTAACAGCTTTCTAACCGAAGTACTGTACGATTTGGCGAATGAACTGCCACGATGAACACTTCTGAAATGAGAAAGATATCGCATAAAATTCAGTTTATCGGCAAGAAGTGTAAATCCTGAATTCTGGGATAGGCCAATTCCATTTTTTGAATTTATGTTGCCGGTTCGGATGAAAAATTCGACTGCAGCGCCAACATCAAATGATCGACCCATTGCCGTATTAATTGCAGCGCTTGAAAGAGTGGAAACAGTCATCGGTTTCGACTGATGAAGAGCAGTCAATCTTGAGTTTAATGAACCCATATACGAGCTCAACTTATCTTTCAGAACCATCAGAATTATATGACCAGGAAGCATGATTTCTTGATTCATTAAAGAATCGGCAGACTCTGAAGAAATTTTTTTATCAACCATGGCTTGCAGCTTTTCAACCATGTATAGGATCAGATTAAATTTGTCCATGTCGCTTTTCAAATGAATGCAGATACAGCGTTCGATAAGAATTTTCGCACATTCTATGTTACTGACAGAATCTGTAACCATGAGTCTACTTCTAAACTTTTCACCGACAAGTTCCAATGTTTTAGTTCTAGAAACCAAATTTGAGACAGCACATTCTGACAACGTGTCTTTGATAGTTTGAACTGCCAAGAGATTATTGTGAACCGCTTTTGACAGAAGAACGAATATTTCGTAATCACAATAATCTGTGAGCATCTTGATAATATAAATGAGAGGAATGAAATATTGACGGTCTCTGAAGACGAAAGAGTACGTGAACGAACCATTTGAGAGATAATGAAGGAAATTCATGTTTTGACTTTGATTCTCATCTACACATCTCAACAGAATTCCATGGTCAGTCATTAATGGAGTCCTTCCTTTCCATGCCGATCTGATGACAGCGATTGGATAATTTCTTCTTTGTTGGATTAACATTCTCAAAACTCGTTCCAAACCATTACAAACGAAGTATCCACCGAATTCGTTGGCTTCTTCATGCATTTTGATTAACTCATTAGATGTTCTTCCATTTAGCCAACAAAGCTGAGACCTGATCATTATAGGAATGTTTCCCAATGGACGAATAATTTCTGGCTGAGAAACTCCATTTACGCTCCAACAAAATCGAGCAGTTATTCTGCCAGCGTAACTTATACCTCTGACTCTGCATTCAGAAGGAGTGAGAATGGGCGAAGATGAGATGTTTTGGTCTAAACGAGGCATTCCAACATGAATATGGATAATTTGAATTTTAACAATAAGATCTTCTTTCAACTTCACAGTCAGAGGTTGGATGTCTTTGAGAGAATTGTTGATACCAACACCAATTGCATAGTTGAATGAGTCCACAAATGATTGAGTGAATTTATTTAAAAACTCGTTTTTAGAATCTGAATTGACCATGTGAACGACAA

**18S** mined from transcriptome

>TRINITY_DN798_c0_g2_i1

AAACAGTGCTAAAATAATCAACAGGAGGGCAAGTCTGGTGCCAGCAGCCGCGGTAATACC

AGCTCCTGTGGCCTATATGGTCGTTGTTGCGGTTAAAAAGCTCGTAGCCGAAATACGGTG

CGCTCGAAAGCTGCATCAGTGCTGAAACTAGCGTCGCCAAGTCACAGATTGTGAATGATG

CCACGGTTAGCCACTAAAACAGCCCGGCACCAGCATCTCGCGACAATAAACTGTGTGCGT

GTGCGTGTGCGTGTTGAATCAACATTCGTACCTTGACTGAGTGTGCCGGCCAGCACGCCG

ACCCCATGCCATCATCACCCTTGGGGGCTGGTGTCACCATGAGACTCGGCACTGAGTCGT

GAGCCTGTGCACACAAGTGCCTGGGGTACGCATCATTTGCCAGCCTAAACAGCCAAGCGA

GCGTCCAATCAGCTGGCTGAGTTTACGCGATTCAGCCCTCATACTTCTTCAAGTGTGTGC

GTGTGGCTGGGCATGTGCGTGCAATTACAACTCGGCATTCGATCGATTGGGCCGAGAGCC

ATTACGCGTGGCTCACGGTGTGCACATCACCCTCAGTTGGTCCTATGTACTAGTATTCCT

CATCTAAGCGCATCACACTGTGCCGTGTAGAATGATGATATGTCGCCGCTGGCTCCTTCA

CTGTGAGCAACGGCCCGTGGCATCTAATCTGCAACATTCGTGCGACGCAATCTATAGTTA

TCCCCGCTGTGGTAACTAACAATGGCTGGGCCCTGCGTTGTGTGCCTCTAACTGTGCGTG

GCCATCACTTCTGGTGGTGGTCACACCGGCATCAGAGCCACAACGCGTCGTCTCATCCGC

CACAGTGGTTCAGCGCTGAGACGGCATTAGAGCGCGCTTTCTCGTCCAGCTCCGGTGCAG

GCACTACGAACAGTAGTGGCGACTATCCTTCACTGGATACACCGGACGCTGAGCGAATTG

TGCCTTGAGTAAAATAGAGTGCTCAAAGCAGGCTAGGCATGTAACAGTGTCATTGCCCGT

ATACTCCAGCATGGAACGAATGTCCAATTGCTTGAACCACCCTGCGACCTGGTTCGTTGG

TTGAAAGTGCGTGTGCGTGTCAATGAGCGCTTCCAGCGCCCAACTCGTGTGCGTTCTTCG

GTACACCCACATCACACCTCATCATGTGTGAGCTGTGGTGTGGGTGGATCCCAGGTCGTA

TCCGCTACGGTGTGTACCCGATGTGCGTGCAGATTCTCGTAATCGCATCAACAGGGCAGT

GTGTGCCATCAGTGGTTGTGTGGTGGCCACCAATGGTCGCCACAGTTATCAATCCGGCAC

ACATTGTGGACACTTTGTCTACTGTCTGCTCCAGGTACTGTGCTGCAGCTGCTTTCCAAT

AAACACAGCCATTAGGTGTGGCTGTGTGGACTGCCAGCCAGCCAGGCACCCAGCGACAGT

ACCAGTGTTACAACCCTGGCTAGGGATCAATACCACCTGTGCGTATGGGTATCGGTTAAC

AGAGACATTTGGGGGCATTAGTACTTGGCCGCGAGAGGTGAAATTCACAGACCGGCCAAA

GACTAACGATTGCGAAAGCATCTGTCCAAGATGTCTTCATTGGCCAAGGACGAAAGTTAG

GGGATCGAAGATGATCAGATACCGTCGTAGTCCTAACTGTAAACGATGCCAACCAGGGCT

CAGGCCGGGTCGGATATGCTGTGTGACGTGTGTCCGTTGGTGCGCCGGGCGCGCCTGTGC

TCAATGTAGCACATGGCTGCCACCCTGGCCCCAAGTGCGTGACAATCACGCTCACACATG

CATATAACAGATCCAGGTTGGGCCCCCCGGGAAACCTAAAGTGTGTGGGTTCCGGGGGGA

GTATGGTTGCAAGGCTGAAACTTAAAGGAATTGACGGAAGGG

**28S** mined from transcriptome

>TRINITY_DN61500_c0_g1_i1

TGAGACGTGAGTCTATCCGGAGCAGCCGTCGGTGCAGATCTTGGTGGTAGTAGCAACTATTCGAATGAGAACTTTGAAGGCCGAGGTGGAGACGGGTTCCACGTGAACAGCATTTGGACGTGGGTTAGTCGGTCCTAAGCAGTCGCCTAACAGCATGTTTCAGGCAAAGGGAGCGGGACCGTTCGCAGGTTCAGCTCCAAGGCTGCGAAAGGGAATCAGGTTAATATTCCTGAACCAAGTGGCGGTTGGTGTGCCTCACCGTGTGTCCGGTGCCGGGCGTGATGTCGCGAAAGCACATCTGCTCGCACCTCATACGGTAGCACACACACATACACGGAGATGCTCTTGGGGTACGTGATGGCGAGAGCTGTCATGGGTCGTTATGTGCGCCCACTGGCGTTCACTCCACAGAGCTAGGCGGTAACGCAAGCAAACTCGGAGACACCCTTCATGGCTCTGGAAAGAGTTATCTTTTCTTGTTAACAGGCCAAGTGATACGCCCCGGAAAAGAGTCAGTCTGAGAGGGGGCTGTGGGCCTGGAAGAGCATCGCAGTTTCAGCGGTGTCCGGTGTGCCATGGTATCGAGGGTCCGTGAAAATCCGAGGGCGCGAGCCACGTGTGTCAAACACATGCGTGAGTGTAATTTCGCACTTGTCCGTACCGAATCCGCATCAGGTCTCCAAGGTGAACAGCCTCTGGCGTTGAAACAATGTAGGTAAGGGAAGTCGGCG

***Myxobolus cerebralis***

**ACTB** from GenBank

>AY156508.2 Myxobolus cerebralis beta-actin mRNA, complete cds

ACGCGGGGAATCTCAATAAAATGACTGACCAACAAGAAGAACAATCCCCAGTAGTTATTGATAATGGATC

AGGAATGGTCAAAGCAGGTTTCGGAGGGGATGATGCTCCAAGAGCCGTTTTCCCTTCTATTGTTGGTCGT

CCAAGACATCAAGGTGTCATGGTCGGAATGGGACAAAAGGACAGTTATGTTGGTGACGAAGCCCAATCTA

AACGAGGAATCCTTACCTTGAAATACCCTATTGAGCATGGAATCGTTACCAATTGGGATGACATGGAGAA

AATATGGCATCACACTTTCTACAACGAGCTTCGAGTAGCACCAGAAGAGCATCCCGTTCTTCTGACTGAA

GCTCCTCTGAATCCTAAGGCTAACAGAGAGAAAATGACTCAAATCATGTTTGAGACCTTCAATACCCCTG

CCATGTATGTCGCCATCCAGGCCGTTCTTTCCTTATACGCATCAGGAAGAACTACAGGTATTGTTATGGA

TTCTGGTGATGGTGTTTCTCACACTGTCCCAATTTACGAGGGTTATGCACTTCCCCATGCTATCCTCCGT

CTTGATTTGGCTGGTCGCGATCTCACCGATTACCTCATGAAAATCCTTACCGAGCGTGGTTATAGCTTTA

CCACCACTGCCGAACGAGAAATTGTTCGTGACATCAAGGAGAAACTTTGCTACGTTGCACTTGATTTCGA

AAGCGAAATGGCCACTGCAGCCAGCAGCTCGAGTCTTGAGAAGAGCTACGAATTGCCTGATGGTCAGGTG

ATCACCATTGGAAATGAACGTTTCCGATGCCCTGAGACCCTTTTCCAACCGTCATTCATTGGTATGGAAT

CCAGTGGACTTCACGAGACACTTTACAACTCAGTCATGAAATGTGACATTGATATCCGTGCTGATCTATA

CTCCAATATTGTTATGTCCGGTGGTACCACAATGTTCCCAGGAATTAGCGATCGAATGAGCAAAGAAGTT

ACTGCTCTGGCTCCATCCTCAATGAAAATAAAGGTTATCGCTCCTCCAGAGAGAAAATACTCCGTCTGGA

TTGGTGGATCTATCCTTTCTTCTCTGTCTACATTCCAACAGATGTGGATTTCCAAGCAAGAGTACGATGA

ATCCGGCCCCTCTATTGTTCATCGCAAATGCTTTTAAACAATAATAGACCTAAAATATATTGCCAGTTTT

AAAAATATTCAAAAAAAAAAAAAAAAAAAAAAAAAAAAAA

**AHC1** mined from transcriptome, from GenBank

>GBKL01003454.1 TSA: Myxobolus cerebralis comp6787_c0_seq1 transcribed RNA sequence

GTTTTTTTTAACAAGTTGTGCATTTTGTTGATTATGATGCATTACAAAGTTGCTGATATT

TCATTGGCCGATACGGGTAGAAAGGAAATTCAAATGGCAGAAGACGAAATGCCTGGTTTG

ATGGAACTTCGACGAAAATATGGGCCGTTGAAAATACTAAAAGGAGCAAAAATCGCTGGA

TCAGTGCATATGACACTGCAAACGGCAGTTTTGATAGAAACTCTTGTAGAATGTGGCGCA

GAGGTTCGTTGGTGTTCGTGTAACATTTTCTCCACGCAGGATAGCGCCGCAGCTGCAATC

GCCAAGGCTGGAATCCCAGTTTTTGCATGGAAAGGGGAAAGTGAGGAAGAGTTTAAATGG

TGTCAAGAGCAAGTTTTAAAATTCGAAAACGGAGGTCCAAATATGATTGTTGATGATGGA

GGCGATTTGACTACTCTCCTTCACTCTAAATATCCTGAATTGCTAAAGAATGTTATCGGT

ATCTCCGAACAAACTACAACTGGTGTGCATAACCTTTACAAATTGTTTAAAAAAGGCCTT

CTTAAAGTTCCGACATTTAATGTTAACGACTCCGTAACTAAAAGCAAGTTTGACAACCTT

TATGGTTGTCGAGAGTCTCTTGTTGATAGTATTAAGCGAGCAACCAACATAATGATAGCC

GGGAAACTCTGTGTGGTATGTGGTTTTGGAGACGTCGGCAAGGGATGTTCAGCGTCGCTA

AGAGGACTAGGTGGGCGAGTGATTATTACCGAGGTTGATCCCATTAATGCACTACAAGCC

GCCATGGAGGGATACGAAGTTACGACTCTTGATGACTGTGTCTCTCGGGCAGACATTTTT

GTTACCACTACAGGATGCACCCAAATTATTACAGGACACCATATTGTTCAAATGAAAAAC

AACGCCCTTCTCTGCAATATTGGCCATTTTGATGTTGAAATAGACACTGCGTGGATTCTC

AAGAATGCAGTTGAACAGGTAAATATCAAACCTCAAGTTGATCGGTTTCGTTTCTCCAAT

GGGAAATTTATTACCTTACTTGCACGAGGCAGACTAGTGAATCTAGGTTGTGCACAAGGT

CATCCCAGTTTTGTTATGAGCAATACCTTTTCTAATATGTTCCTCTCCCAGGTAGAGCTA

TTCACTGCGCCTCCAGGGAAATACCCCGTTGGAGTTCATTTCTTACCGAAATTGCTCGAT

GAAGGAGTTGCTTCTGCTCATTTGGGAGCTCTGGGTGTTAAACTGACTCACCTTACGCCG

GAACAATCTCAGTACATCGACGTCCCACTAACGGGACCATACAAAGCTGATCATTATCGC

TATTGATTGATTTATTCGTCTACAATATTTTTATTCGAATAAAAAAAAAA

**EF2** mined from transcriptome, from GenBank

>GBKL01021688.1 TSA: Myxobolus cerebralis comp1023_c0_seq2 transcribed RNA sequence

GAAGGATTTTCCGAAGCAATTGATCTCGGAAAAATTTCACCTCGTGATGATTTGAAGGAGCGGGCCAAGT

ACATGAATGAAAACTTTGGCATTCATCCAGAGGAAGGCAGAAAAATATGGTGTTTTGGTCCTGATGGATC

CGGGCCTAACCTTTTGATTGATGTCACAAAAGGAGCACAATATCTCAGCGAAATCAAAGACAGTTTGGTG

GCAGGATTCCAATGGGCATCCAAGGCAGGTCCTTTGTGCGAACAAAACATGCGGGGTGTTCGTCTGGACT

TGCATGACATCACTCTTCACGCTGATGCTATTCATCGTGGAGCTGGTCAGCTTATCCCTACAACTCGTCG

AGTTACATATGCAGCCGTGCTTACTGCTGAACCAAGAATTGTTGAACCCATTTACTTGGTCGAAATAATG

GTACCACAAGAGCATGTCAGCGGAATCTACGGCCTCATGAGCAAGAGAAGAGGTCATGTGTTTGAGCAAG

AATCGGGCATGGGATCTGCTCTCCATATTAAAGGACATCTTCCAGTCAATGAATCGTTTGGTTTTACTGC

TGAAATTCGAGCAGAAACAAGTGGCCATGCATTCCCGCAGTGTGTCTTTGATCATTGGAAAATTATCCCA

GATAGTCCGTTTGATTCTACCACAAAGTCAGGTGCGATTGTTGAAGAAACCAGACTTAAAAAGGGTCTAA

AAGCAGGAATCCCTGCACTAGATAACTACCTCGACAAACTCTAATTTATATTTTTCTTTAATTTTACAAA

TATTTGTTATTCTTTCAAAAAAAAAAAAAAAAAAA

**GAPDH** mined from transcriptome, from GenBank

>GBKL01017634.1 TSA: Myxobolus cerebralis comp118_c0_seq1 transcribed RNA sequence

AGAATGTTCTAGCAAACACGCCGTACCGCATAAAGTATAAATATATAGAACCCAGTCTTTAATCCATTAG

CAAATTTATTTGTTTAGAAATGACGGCAGCAAAAGTTGGAATTAATGGTGGTGGTAGAATCGGACGAGTT

GTTATTCGACAAGTTTTGGACGCTCTTCAAAAAGGAGACAATTCAGTACATATCATGGCAGTCAATGACT

TGATGATTGCGCCAGAATACATGGCCTACCTTATTAAGTATGATACAACACATGGAGTTCTTCCAATGGA

TGTTAAGGCTGTTGGTGATATCGTTATGATTGGAGATTTGATGAAATTCAAAGTAATAAAGGAAGCCGAT

CCTAGCAAAATCAATTGGGCCCAACTTGGTGTTGATTACGTCATCGAGTCAACCGGTCGTTTCACAAAGC

ACGATCAAGCAAAAATGCATTTACAAGGCGGAGCCAAGAAGGTTATCATTAGCGCACCTTCTGAGGATAG

CCCTATGTTCGTATTCGGAGTTAATCACCAAACTTATGACCCAAAATCAATGGATGTCATTTCCAATGCC

TCATGCACAACTAATTGTTTGGCCCCTCTTGCAAAGATTATCCACGATAGCTTTGTAATTGAGCAGGGAC

TTATGACGACCGTACACGCAATGACCGCTACACAATTGGTAGTTGACGGTCCTTCGGCAAAAGATTGGAG

AGCAGGAAGAGCTGCTTCTGCCAACATTATTCCTGCCTCCACAGGTGCTGCTAAGGCTGTCGGAAAAGTT

ATTCCCGCTTTACAAGGAAAGCTTACTGGAATGGCTTTCCGATCACCAACAATCAATGTATCTGTTGTTG

ATCTCACTGTTGTGGTGGCAAAACCCGCAACTAAACTCCAAATTGATAACGCAATCAAGGCTGCTGCTGA

CGGGCCCATGAAGGGAGTTATCCAGTTTGTCGACGCACAGGTTGTGTCTTCTGATTTCAATGGGTGCCCA

TTTTCATGCAGTTACGATTCGTCTGCTATGATTACTTTCGATCCAAGCAAACCACAAACCATGTTTAAAT

TATGTGGATGGTACGACAATGAAACTGGATACAGTGCACGTATGGTGGATTTGATAAAATTCATGCATAG

CAAGCATTAAATTACTATGATAAATTAATTGGCTAAATTATTAAATCTTGCTTTTGATAAAAAAAAAAAA

AAAAAAAAAA

**HPRT1** mined from transcriptome, from GenBank

>GBKL01050483.1 TSA: Myxobolus cerebralis

ATTAACCATTATTAAGCAACGTGATCAGTCGACTATCTATAAATATTCTTACCAATTCGTGTAAATAATC

CGATAATAAACATTTTGTTTTCGATGATGCATGCAAAAAAGAATACTTATAATCCTTTGGTCTTTCCCGA

TAGCTTTAATGGTTACCCAAAAGAACTGTTTGGTGCTCCTGGTGAAGAAATGAAACACATCGACCACTTG

TTGATTCCTCGTGGTCTGATCAAAGATAGAGTTGAAGTTGTTGCTAGGGAATTTTGGGAAAACTGGGATG

GACGACCTCTACTTGCACTGTGTATCATGAATGGAGCTGCTCGATTCTTTGAACTTTTCGTTCGTTGTCT

CAATAAATTAAACGTCCAATCAGAACAAAAAAAGCATTTTGTTATCAAATATTTGAAAATGGAAAATTAT

ACAGGTGATCAGCCGACGCATGCTCCCATAAAATTACCAGAGTCTTTAATCAAATCTTTGGCTGGAAATC

ATATTCTTGCAGTTGAAGACATTGTTGATACCGGGCATTCTATGGTTAAACTTATCGAAACCTTTTCCAG

ATACGATATTTTGTCTTTTCAATGCTTGAGTATGACCACGAAAAGAACTTTAAGAAGTAATGGCTATAAA

CCTGATTATTGTTGCGTTGAAATTCCAGACCGCTGGGTGGTAGGCTTTGGACTAGATTTTGATGATGAAT

TTCGTCATTATGATCATGTTTACGTTCTTTCGCCTAAAGGAATTAAAGATCTAACCAAATAATCAATGAC

TGAATTGACATGACTTAGTGCTATTTATCTATATCCTTTACCAAAAAA

**RPB2** mined from transcriptome, from GenBank

>GBKL01027608.1 TSA: Myxobolus cerebralis comp10463_c0_seq1 transcribed RNA sequence

ATCTACCCAAGCGAATGCCGAATCAAAGGAACCACTTATAGCGGAAAACTAATGGTACGT

TTAGGGTGGTCCCTAAATGGCGAGTACCAAGGAACTGTCTCGAGAATACTTGGCAATGTG

CCTGTTATGATCAAATCTAAACGCTGTTCGATACAATCATTAAACGCACATGAATTAATT

CGACACGGCGAAGAAATTGATGAACATGGGGGATATTTTATATGCACTGGTATAGAGCGA

ATTTTACGATTATTGATAGTTCAACGTCGAAATTATCCATTGGCTATCGTGAGACCATCT

TGGAAAAATAAAATGTCAGGTTTCACGGAATATGGTGTATCTCTGCGATCAGTTGATGAA

TACAACAATGGAGTGACTAATATAATGCATTACAATAGCAATACGACTATTGTTTTGTCT

CTTTCTTATAAGAAAAAGCAATATTACATGCCTTTTTTTGTCGCACTTAAGTTACTCGTT

AATTATAGCGATTTCCACATTTACTCCGATTTATTGCTCAATAATGCTGAAAATTCAGTT

TGGATGGAATCCGCAAAATGCATGATGTCAGTAATGCACAAAATGAAAATCTGCAAAACG

GACGATGCTAAACGACATGTAGCCGAAGCCTTTCGAATAAAACTTGGATTACCAAATTGG

GCAAGCGCATCTCAAGTCTCTGATTTCCTCTTAAAAAACTGTATATGTGTCCATCTCAAT

TCGAATGTCGACAAGTATAACATGCTCATATTCATGGCCCATAAGTTAATGGCCCTTGTT

GATAATAAAATATCACCTGAATCAACTGATACTCTGAATAACCAGGAAATTTTATTGCCC

GGTCAAATTTATCAAATTATATTACGTGATAAAATGGAGGGCTGGCTAAACGCTTTGCAA

TACAGGATCCAAGCTATGTTTAAAGTTGATTCCTCCAGTAAAATTTTTACGCTTGCCCAT

TTAAACACGGCCATGGGACGGTGCCCTGACATCGGATTACAAATGGAATATTTTGTTAAA

TCCGGCAACGTTGTTTCTAAAAGTGGATTAGGATTGTCACAGGTTACTGGATACTCGGTT

GTTACCGATAAACTAAATTATATTCGATATTTGTCACATTTTCGCTCAGTTCATCGTGGC

GCTTATTTTTCTAAGTCCTACAGCACGAAAGTTAGAAAGTTATTACCTGAATGTTGGGGC

TTCATGTGCCCTGTTCATACCCCCGACGGATCTCCCTGTGGGCTATTAACTCATTTAGCT

CAATCCTGTGAGATTGTAAGTAAGGAGATTGAGGTTGAGCCATATTTAAATACGGTTTTC

AGTGCTGGCGTTATTCCATGTGTTCCTGGCGCCAATTTATCAAATAAAATTGTTATTCAA

ATTGACGGGAAAATTGTTGGATACATTGATTCAAATATTGCAGAAAAATTAGTTTCGAAA

TTAAAAGATTTTAAAGTTAAAAATCTACATAATATTGAAATTGTTTTTGCTCCCAAAACT

AAAGCTACTCCTCTTTATCCTGGTATTTTCATATTCTCCAATCCTTCTCGATTAATTCGT

CCTGTTTTCAACTTGGAATCAAAAACTATTGAATATATTGGTCCGTTTGAACAAATTTAT

TTATATATTGCTATCTCTGATAAAGATGTAAAACCAGAAACAACACATAAAGAAATCGAC

CCCGTTTCTATTTTGAGCATAACTGCGTCGCTAACTCCTTTTTCTGATTGTAATCAAAGT

CCTCGAAATATGTATCAATGCCAAATGATGAAACAATCAATGGGTGTAGCATTAATGGCC

CACCGTTATCGATTTGATAATAAGCTGTATCGGCTGTTGAGTATACAAACACCTATTTGC

CGAAGTTTAACATATGAAAACATTCAACTCGATAATTACCCTATCGGACTGAATGCAGTT

TTGGCGGTAATTTCATACACGGGTTACGACATGGAAGACGCAATGATTCTTAATAAGGCT

ACAATCGAACGCGGTTTTGCTTCTGGTTGCATGTATAAGTCCGTACTTGTTGATTTAATC

GAAGAAGCCGGAAACAGCAAGGATCGTGTTTTGATTTTTGGGTGCAAAAATACCGATAAA

TGTTTTTACGAAAATCTTCTTGATCTTGATGGGTTACCACCTGTTGGCCGTTTTATTTCA

CCTGAAGAGACTTTTTACAGTTATTATGATTTACACACTTCTGAATATACTGTAAAGAAA

TTTAAAATTTCTGAGGGTGGTTATATTGATGATATCGCAATTATTCAGTCAGATCAATTT

GGTCCCCGAAAAGCAAATATACGTGTACGTATTCCGAGGAACCCGATAGTGGGAGATAAA

TTTTCTAGTCGGCACGGACAGAAGGGAATTTTGAGTATCACCTGGCCCGCTGAAGATATG

CCCTTCACAGAAAGTGGCATCATTCCTGATATAATTTTTAACCCACATGGATTTCCGTCT

AGAATGACTATTGGGATGATGATTGAAACAATGGCTTCCAAAGTCGGGGCCTTGAATGGT

AAATTTTTCGATTGCAGCCCGTTCAAGTCCGAAGATAACTCTCTAGTTGATTTTTTTGGT

CAACAACTTTCTGATGCTGGATATAATTATTACGGATCCGAACGATTGTACTCTGGAACT

AATGGTGAAGAATTAGAAGTTAATATATTTACCGGTATTGTTTATTATCAACGCTTGCGC

CATATGGTTTCTGACAAATTTCAAGTTCGCAATACCGGTCCTGTAGACGCGCTCACTCAT

CAGCCTGTTGGTGGGCGTATCAGAGGTGGTGGTGTTCGTTTTGGAGAGATGGAACGTGAT

GCTTTGATTTCTCATGGTTGCTCTTTTCTACTTCAAGATCGACTTTTTGATTGTTCTGAT

AAATCACTTGCATTTGTTTGCGAAAAATGTGGTGATATTTTAGCGACCAAATTGAATCCA

GATAGAAGCTTTAATCCTCTTGTTAATTTCAAACCTCCTTCAGTTAGTGAAAATAAAAAT

TGTAAACAATTTATTTGTCTATTGTGCAATTCAAGTGCTCACGTTAAACCTATTTATATT

CCCTATGTATTCCGTTATTTCCTAGTCGAGCTTGCCTCAATGAACATCCAAATTAAACTT

AATTTATAATTATTATTTTTTTGTATTTTATAAATTTCAAAAAAA

**18S** from Genbank

>EF370479.1 Myxobolus cerebralis 18S ribosomal RNA gene, partial sequence

ATATACGCTTTTCTCTAAGACTAAGCCATGCATGTTTAAGTTCATACGTAGTAAAACGTGAGACTGCGGA

CGGCTCAGTAAATCAGTTATCATCTATTTGATTGTCTACCCATTGGATAACCGTGGGAAATCTAGAGCTA

ATACATGCAGTTTTGGGACAGCGTTAAAACTGTCTCACGGCATTTATTGGACTAAACCAACTACCGTTGC

ATTGGTTTACGCTGATGTAGCGAGTAAGGTGAATCTAGATAACTTTGCTGATCGTATGGCCTAGAGCCGG

CGACGTTTCAATTGAATTTCTGCCCTATTAACTAGTTGGTAGTATAGTTGCCTACCAAGGTTGCGATGGG

TAACGGGGAATCAGGGTTCGATTCCGGAGAGGGAGCCTGAGAAACGGCTACCACATCCATGGAAGGCAGC

AGGCGCGCAAATTACCCAATCCAGACACTGGGAGGTGGTGACGAGAAGTACTAAGTGGTGGCCCTTAGGG

TCGCCAGCTTGGAATGGACGTAATTTAAGTAATTCGATGAGTAACAACTGGAGGGCAAGTCTGGTGCCAG

CAGCCGCGGTAATTCCAGCTCCAGTAGCGTATTTTAAAGTTGCTGCGTTTAAAACGCTCGTAGTTGGATC

ACGCAGTGTAAGTTGGTAGGCTGATCGAATGGTGCTACTAACTGCTCCAGCGTTGAATTTCAAATTCAGT

GTTGGAGTAGTGTGCCGTCTTTCAGTTATTCGCCAATTTACACTACTTACGCGTAAGGATGGCAGTTGAC

CTTTAGTGCGTCGATTGCCGTGTCTTACGGAGTGTGCCTTGAATAAATCAGAGTGCTCAAAGCAGGCTTT

TGCTTGAATGTTAATAGCATGGAACGAACAATTGTGTAGTAGTGTGTTGTGACGAATAGCGATCGGTCTT

TGACTGAATGTTATTCAGTTACAGCATACAGCACCAACCACCAATAACGGATGTTGGTTCCGTATTGGGG

TGATGATTAAAAGGAGCGGTTGGGGGCATTGGTATTTGGCCGCGAGAGGTGAAATTCTTAGACCGGCCAA

GGACTAACGAATGCGAAGGCATTTGCCCAGACCGCCTCGCTTAATCAAGAACGATAGTGGGAGGTTCGAA

GACGATCAGATACCGTCCTAGTTCCCACTGTAAACTATGCCGACCCGGGATCAGCATGAAGCTCTTTATA

CGCTTTATGTTGGTCCCCCTGGGAAACCTCAAGTTTTTCGGTTACGGGGAGAGTATGGTCGCAAGGCTGA

AACTTAAAGGAATTGACGGAAGGGCACCACCAGGAGTGGAGCCTGCGGCTTAATTTGACTCAACACGGGA

AAACTTACCAGGTCCGGACATCAATAGGATAGACAGACTGATAGATCTTTCTTGATATGATGGATAGTGG

TGCATGGCCGTTCTTAGTTCGTGGAGTGATCTGTCAGGCTAATCCCGGTAACGAACGAGATCTTATTCTC

CATTTGATGAGCGGAAGAAGATAGTGTAGCTCGATGATTGTTTCGGCGATTCTCAAGTTATTCTATCGTA

GGCAGTGTTTGTGAATTCAGCGTGAAAATACAGTTTGTTGCGAGGACGGGATAAAACTCTTACTTGTTGC

AAATTGTACTACACCTGAGTTTGTTGGCATTCCCTTCCGTTATACGCTGTTCAACTACCCAGTTGAGCAG

TGTGTCATGGAGAGACTGTGAGGTATATATCCAAGCTCAATGAAGCAAGGCCATAACAGGTCTGTGATGC

CCTAAGATGTCCTGGGCTGCACGCGCGCTACAATGATGGTGACAGCGAGTTTCTAGGTCGAGAGACCTGG

GCAATCTTGTAATCACCATCGTGATGGGGATTGACCATTGTAATTTGGTCATGAAATAGGAATTCCTTGT

AGGCGCACTTTATTAGAGTGTGCCGAACGAGTCCCTGCCCTTTGTACACACCGCCCGTCACTACTACCGA

GTGAATCGTGTCATGATGCCTTGGGACCGGACGTATTTGGAGCTGCAAGGCTTGAAATACGCTGGGATCG

ATGTAAAATGGTGCAATTTMGAGGAAGTAAAAGT

**28S** from GenBank

>AY302740.1 Myxobolus cerebralis 28S ribosomal RNA gene, partial sequence

ACCTCCACTCAGGCAAGATTACGCCGTGAACTAAAGCATTTCAGTAACGGCAGGAAAAGAAAATAACTAT

GATTCCCTCAGTAACTGCGAGTGAAGTGGGAAGAGCCCAACGTTGAAAGCTACATCTTTAACCGGGTGTC

GCGTTGTAACGTATAGATGCAACATCGAGACGTGAACCAGATTTGAAGTCGCTTAGAATAGCGCACCATA

GAGGGTTTTAGTCCCGTACATGAATTTGTGTGTCACATCAAGTATGTTGTCTTCTAAGAGTCGGATTGTT

TGGGAATACAGTCTGAAGTGGGTGGTAAACTCCATCTAAGGCTAAATATAACCTCGAGACCGATAGCGAA

CAAGTACCGTGAGGGAAAGTTGAAAAGCACTCTGAAAAGAACGTGAAAAGGGCGTGAAACCGTTAATGGG

GAAGCGTACGGTAGAGTCGAAGTAGAGCAGCGTGGGTGCGCGTATTTTGTGGCAACACAGGATGCGTGGC

AGGTAGCGTTACTCTACGCGCCGGACTCATTGTTAAATGACTGGCGTAGTAAGTGGTTGGTAGTAACAGT

GCATCCCTGAGGATGTTCTGTGCTATTAGCATTTACAATATGTTTTAGAGTTGTTCGACAATGATGTGTA

GAGCGTAATATCAATGTGTAGTGGCTATCGGTAGAGTGCAAGTTTAGGCAAACACTCTATTGACAACCAT

TTCGTATTGGTGTTACAAGACAAGTCGCATACGATTTTCCGGCGAGACATGCTACTGTTCGACCCGTCTT

GAAACAC

***Ceratonova shasta***

**ACTB** mined from transcriptome.

>TRINITY_DN23296_c1_g4_i1 len=459 path=[1833:0-300 1834:301-336 1835:337-458] [-1, 1833, 1834, 1835, -2]

GGAAAGTTGACAATGATGCTAAAATTGATCCTCCAATCCAGACAGAGTATTTTCTTTCAGGTGGAGCAATAATCTTAATTTTCATTGTTGATGGGGCCAATGCAGTCAATTCTTTTTGTACTCTGTCGGCAATTCCTGGGTACATAGTGGTACCACCAGACATAACAACATTTGCATACAAATCTTTTCTAATATCGACATCACATTTCATGATGGAGTTGTAGGTAGTTTCGTGAATACCAACAGCTTCCAATCCTAAAAATGCCGGTTGGAAAAGAGCTTCTGGGCATCTGAATCTTTCGTTTCCAATGGTAATAACTTGACCATCAGGAAGTTCGTGACTCTTTTCAATGCTTGAACTAGAAGCAGCAGTTGCCATCTCTTGTTCAAAGTCCAAAGCAACATAAGCCAATTTTTCTTTGATGTCTCTAACAATTTCTCTTTCAGCTGTAGTGGTGA

**AHC1** mined from transcriptome

>TRINITY_DN30270_c0_g1_i1 len=1429 path=[1407:0-1428] [-1, 1407, -2]

TTTGAGCAAAAAAAATTAAAATCTATAAGATTAATATCTATAATATTAATATCTATAAGATTCGGACTTAAAAGGTCCCTTGGTACTAACATTAATATAATCAGCTTGTTTTTCTGTAAGAGTAGTAAGTTTGACACCAAGCTTTGACAAATGCAGACTAGCAACTTTTTCGTCTAGGTATCTAGGCAGAAGATGTACACCCACGGGGTATTTCCCCTGATTTGTATACAATTCAATTTGAGCCAAAACCTGGTTACAGAAACTGGTACTCATGACAAAAGAAGAGTGTCCTTTGGCACATCCTAAGTTAACTAACCTTCCTTCCGCTAAAACAAGAATTGAACGACCATTTTTCAGTTCAAAAAGATCTACTTGAGGCTTAATAGTAATTTTTTTAACGTGATTCTGGTTTAGCCATGCCATATCGATTTCAATATCGAAATGACCAATGTTGCATACAATTGCGTGATCCTTCATTTTCAAAATATGATCACCATTAATTATATCACGGTTCCCTGTACAAGTAACAAATATATCCCCAGTAGAAGCTATTTCATCCATAGTTGTGACTTGAAACCCTTCCATTGATGCTTGAAGTGCATTTATTGGATCAATTTCGGTTACCACGACTCGGCCTCCGAGCCCCTTAAAGGCTTGAGCGCATCCTTTACCAACATCACCATAGCCACCCACTACACAAACCTTTCCTGCAATCATTACACCAGTAGCTGACTTTATTCCATCCATCAGTGATTCTCTGCATCCGTAAAGGTTATCAAATTTGCTCTTTGTAACAGAGTCGTTGACATTTATAAAAGGAAGCTTTAAATTTCCTTCCGATTGCCATTTTTTCAAACAAAATATTCCGGTAGTAGTTTCTTCGCTTCCCCCGTAAATTCCAGAGATTAGCTCTGGAAATTTTGTATGAACCATTTTTGTTAAATCGAATCCATCGTCTAAAATCAGGTTCAAAGGCATAGTCTTATTTGGACCAAAGAAAATAACTTGTTCGATGCACCAAAGGAATTCTTCCTCTGTTTCTCCCTTCCAAGCGAAAATTGCAAGTCCACTTTTAGCTAGAGCAGCAGCAATGTGATCCTGTGTAGAAAAAATGTTGCTAGAAGCCCATCTTATCTCAGCACCCAATTCAATTAGAGTTTCAATGAGAACAGCAGTCTGTACGGTCATATGAAGGCATCCACCAATTTTAGCACCTTTCAAAACCTTTTTGGGTCCAAATTCCTTTCTTATTTCCATTAAACCAGGCATTTCTTTTTCTGCCAACATAATTTCTTTCCTTCCCCATTCAGCTAAGTTTATATCTGCAACCTTATAACTTAACGCCATTATTTCGTACTTAAAATTTGAGTCGTAAACAATAACATATGCTTAAAAGATTAATGAAATGACGTGGTTATTTCAATAACTT

**EF2** from GenBank

>KM392431.1 Ceratonova shasta elongation factor 2 (EF2) gene, partial cds

GGGGAATTACATTTGGAAATTTGTTTAAAAGATTTAGAGGAGGATCATGCAGGTATTCCAATTAAAAAGT

CCGACCCGGTTGTATCTTACAGAGAAACTGTTACAGAAGATTCTAGTGTTGTTTGCTTATCAAAGTCTCC

TAACAAGCATAATAGATTGTATGTTACTGCTCATCCTATGCCTGAAGATTTACCTAAAGATATTGATAAT

GACAAAGTTTCGCAAAAGGACGAAGTTAAAGCTAGAGGAAGATATTTACACGAAACATATGGTATGGATC

CAGATGAGGCTAGGAAGATTTGGTGTTTTGGACCTGAAACTACAGGACCTAATATATTAACCGATACTTC

TAGAGGAGTTCAATATTTGGGTGAAATTAAAGACAGTGTTGTTTCTGGATTCCAATGGGCAACTAAAGAA

GGTCCTATGTGTGAAGAAAATATGAGAGGAGTTAGATTTGCTATTAAAGATGTTGTTTTACATGCTGATG

CTATTCATCGCGGAGGTGGTCAAATTATACCTACTGCTCGAAGAGTTATTTATGCTTCCTTGTTAACAGG

TCAACCTCGTTTGTTTGAACCAGTATATTTAGTGGAAATCCAAACTGATGAAAGATCTGCACAATCTATT

GGTGGAGTAATGACTAAAAGAAGAGGTATGGTATTTGAACAAGAATCGATTAATTCTTTCCAATGTATGG

TAAGAGCATATTTACCAGTCAATGAAAGTTTTGGTTTTACTGCTGCATTACGAGAAGCTACTGGTGGAAA

AGCTTCCCT

**GAPDH** mined from transcriptome.

>TRINITY_DN29264_c10_g2_i1 len=1930 path=[5612:0-1929] [-1, 5612, -2]

TTTTTTTAACAAAAAAAATAAAATAAATTTAAAATAAGATTTTTACTTTTTGCTAATATCTTGAATCAGATCAACCATTCTGTTGCTGTATCCCATTTCGTTATCATACCAAGATATAATTTTGAAAAAAGTTTCTCCCATTCCCATACCAGCTTTACTATCAATAGTTGATGAAAATGTTGTTCCGTTAAAATCAGAAGAAACAACTTCTTCATCAGTAAAATTCAAAATTCCTTTCAAAGCTCCTTCACTTGATGCTTTCAAAACTTTCATAATTTCATCATAAGTGGTCGGTTTTGATGTCCTGAAGACAAAATCAACAACTGAAACATTTGCTACTGGGACTCTAAAAGCCATACCAGTAAGTTTTCCATTTAATTCTGGTATAACCTTTCCTACAGCTTTAGCTGCTCCTGTTGATGCAGGAATAATATTGCTATAAGCATTTCTTCCATCTCTCCAACTTTTCATTGAAGGTCCATCGACTGTTTTTTGTGTTGCCGTAACAGCATGAACAGTTGTCATTAAACCTTCGATAATTCCAAAATTATCGTTAACAACTTTTGCAACTGGGGCTAAACAGTTGGTGGTACATGATGCATTACTGAGAACATTCATAGAACTATCATATTTGTCATTGTTTACACCGTAAACAAACATAGGGGCGTCTGGGGAAGGAGCAGAAATAATTACTTTCTTAGCGCCTCCTTTCAAATGTCCACTTGCAGCTTCAACTGAAAGAAAAATTCCTGTACTTTCAATTACAAAATCTGCACCTAATTTTCCCCATGGAATATCACATGGATTTCTCTGGGTCTCAAACTTTATTTTGAAAACATCTTCGATGGTAACTGATTCATCATCATAAGTAACATTATTCTTAAGCTGTCCATGAGTGGAATCCTTCTTCAACATGTAGGCAAAATATTTTGGACTCATAAATGGGTCGTTAACTGCAACAATGACAACATTCTTATCGTTGTTAGCATATCTTTCAACAATGGCTCTTATAACACATCTGCCAATCCTGCCAGCTCCGTTTATACCTACCTTAACAACCATTTTATCTTCGTTATCGAGATTAGCAAACGAAGATTTTTATATTAAAGAATTAAGTCAAATCCTATTGACACATACAAATAATTGTTTTTATCTGAATAGATATCTTGTATAGAATAAAATTTCATTTAATTTAGGAAACAATAATCTTAATCGTTTTTCGTAGCTGAAATAAACAATTATTCGATAAACCGCAAATCACACACAATCGTCTTAAATAAATTTTAAAATATATTATTCTCGAACAAAGCTTTTTCTTCATCATCTACTTTGAAAATGTAATCGAGATTATAACGAATGGTTTCAAAAAAGGATTTTTTTTCGCCATTTTCTATTTTTCTATTCTTAGTAGGTTGCTTAGTAACATATTCCAACGTGTATATAGGTAGAAATGAATCGATAACCCCGCAATCCTCTGTTAGGTTGGGAAAGAGCCAAAATTTCCATTTGAAAAAGGATAACACAAAGATCATACTAAAGATGATGTATCTGACGACTGCAAGGGCCATGAAGACAACCAAAAGCGCAATCAAGGCGTAAAAACCATATTGGAAAGCTTCCTGGGCATTTTTCGGCCAAATAGGAGTTAAATTGTAAAATAAAATTGCGACAACGAATGCAATTGAAATTCCGTAGCTTGCAAGTGTATTTACCGGTTTATAGTTCCAAACGTAAAAATCATTTTCAGAATCTTTAAAGGTCAAATTATTCATATTAATAGTCGCCAACTTTTTAAAAATTAAGTTTTTAATTTCTTCCTCAGAATCATTTTTGTTAATACATGAGATTTGCTTTAATCTAGAATTCTTGTTGGCTCTTTTAAGATGAAGAAATTCATTTGTATTAATTTGTTTCTTTCTTAAATTGATGG

**HPRT1** mined from transcriptome.

>TRINITY_DN5718_c0_g1_i1 len=935 path=[1915:0-565 1916:566-934] [-1, 1915, 1916, -2]

AATAAGTTATTTTTGTAAATATTTTTTTTGTAATCAATGTGTTAATGGTAAAAGAAGTTATAAACAATTTACCTGTATTATGATAATTCGTATTAAAATTAAATTTATTTTTTTAATAATTGATAAAAATATATATCTGTATTTTTAATAATATTAAGTTTTATAATCAGCTTAAATAATCTTCGGATCTCTTATCAATTTATCCTTTCAATATAATGAAGTTGACATCTAGAAAACTTAAAAGTTCTGATACTTTAAGTATTCCAGATGAATTTGAAGGATTTCCAAAGGAGTTTTTTTTAGGTCTAAATTGTGATCCAAAATATATTGATCATATATTAATTCCAAATGCTCTGGTTAAGGAAAGAGCAAAAAAAGTTGCCAAAGAACTTTGGGATTTCTTGGATGAAAAACCAATAATCATGTTATGCGTGTTGAAAGGAGCTTCAAGATTTTTTGAGATATTTACACAATCGTTTAACTTGGTTAATGATTATCAATCTAAACCAAAAGAGTTTAGAGTTGATTACTGTAGAACTAAAAGTTATGATTTTAGTATTCAAAATAACAATTGTCAACTTTCGATGATTGAAGAATTGGTAATTGCAGACAATAATATAGTTATTATTGAAGATATAGTAGATACTGGTAAATCAATGATTGAATTGATTAAAAATCTGAAATGTAAAAATCCTGCTTCCATTACAGTTTTAAGCATGGTTTTAAAAAGGAAGGAAGGTGTTCTATTAGAACCGGATTTTTCATGTTTTGAAATTCCAAATAGATGGATTGTTGGTTTGGGACTTGATCATAATGAAAAACTTCGTAATTTGGAACATATATGTGTATTATCTAATGAAGGTATTAAACACTACAATGAATGTTTGTAACAGTCTTTTTTGTTTTTATTATAAAAAAAAGATACAAAATTTAAT

**RPB2**

mined from transcriptome.

>TRINITY_DN29346_c0_g1_i1 len=923 path=[901:0-922] [-1, 901, -2]

AGATTGTTTGATTCCAAGATCGTCATAATTAATTGACAATTTTAACGTTTCATCAGTCAAATCTAAATAATAATTAGTTATTTTGCAAATTTACACGTAAAATAAAAAATAATATTTTACTTTTAACTGATTCCTTCAATAAAGAAAACATCCTTCTGACAATTTTCAATTTAGTTAACGAAAACAAACGAATTAAAAAAACAACTCATCGTCGAATTTAAAGACGATTCATGTTTTACATAAAGACTCTAAAAAAGGACGTGTTAGTACATCCAAAATATTTTGGACAAGGAATAAATGATACTATAAAACAAAGGCTTTTAATGGAGGTTGAAGGTACGTGTAATGGAAAATATGGTTATACAATTGCCGTAAAGAATATATTAGATGTTTCAAATGGTGAGTTAGAGCCAGGACTTGGTTTTGTAATGTATAGAATAAAATACGAAGCTTTGATATTTCGTCCTATAAAGGGGCAGATAATTGATTTTTGTGTCTCTGGAATAAATAAACATGGAATATTTGCTGAAGCAGGTCCATTATCTTGTTTTACATCGAAACATTGCATTCCAAATGATTGGAACTATGAAGCAAGTAATCCAAGTGATCCTTGTTATATGTCAAAAATAACTCAAGCAACAATTAAAAAAGGTGATTATCTAAGAGCAAGAATAATAGGTATTAGAATAACATCAAAGGAAATGTTTGCAGTAACGTCTATAATGGAAGATTTTACCGGCAGAACCATATAAAGTATGTACCATAGAATTTTGTTTGTTTAATATTAATCTCCGATTTTACATTGCTTTATCCAATAAAAATAATTTTATAAGAATTATTTTAAACTAGTGAATCATGTACTGATATGGTTTGCTTTTTTTTTACATAAGGGGATTTTAGTTGTATCTAAAAACTACTATTTT

**18S** from GenBank

>AF001579.1_C_shasta_18S

CTGGTTGATTCTGCCAGTGGTCATATGCTCGTCTCAAAGATTAAGCCATGCATGTCTAAG

TTCATATCATTTAAATGATGAAACTGCGAAGGCGTCAGTAAATCAGTTATCGTCTGTTCG

ATCGATACATGCCATGGATAACTGTGGCAAACCTAGAGCTAATACATGCAAAATTCTTTG

TTTCGGCAAAGAAGCATTTATCCAGCTAAACCAATCGGGCCTTAAAACCCAGTAGGTGAA

TCTAGATAACTGTGCCGATCGTTGCTTTATGCGGCGATATTTCGATTGAGTTTCTGCCCT

ATCAACTTGTTGGTATGGTATTGGCCTACCAAGGTTTTGACGGGTAACGGGGAATCAGGG

TTCGATTCCGGAGAGGGAGCCTGAGAAACGGCTACCACTTCTAAGGAAGGCAGCAGGCGC

GCAAATTACCCAATCCAGACATTGGGAGGTAGTGACGAGAAATACCGGACTGGATCTTAC

GATCCAGTACTGGAATGAACGATATGTAATCATTTCGATGAGGATCTACTGGAGGGCAAG

TCTGGTGCCAGCAGCCGCGGTAATTCCAGCTCCAGTAGTGTATATCAACATTGTTGCGGT

TAAAACGCTCGTAGTTGGATAACGAGGGTAAAATAATTATATGAGCAAATGCTCATTGAT

TATTTTGCTCTTTTTATTACAAGGGTCAATACTTTGCTTAATTGAATTGTATTGAATACT

TGTATAGCGTGCCTTGAATAAAGCACAGTGCTCAAAGCAAGCGTAACGCTAGAATGTTAT

AGCATGGAACGAATAGATTGACCTGAATCAGTTTGTTGGTAAAAAGTTACGCAAGTAACT

AACCTGAAACGGGTCCTGATTAAAAGGGGCATTTGAGGATGTTAGTACTTGGTGGCGAGA

GGTGAAATTCTTAGACCCACCAAAGACTCACTATTGCGAAGGCATTCATCAAGAATGTTT

TCATTAATCAAGAGCGAAAGTTGGAGAATCGAAGACGATCAGATACCGTCCTAGTTCCAT

ACAGTAAACTATGCCAGCTTGAGATTAGCTCGGTAAACGAGCCAAGTTGGTCTCTCCGTG

AAAACAAGCTTTCGGGTTCCGGGGGGAGTACGGTCGCAAGTCTGAAACTTAAAGAAATTG

ACGGAAGGGCACCACCAGGAGTGGAGCCTGCGGCTTAATTTGACTCAACACGGGGCAACT

CACCAGGTCCGGACATTGAAAGGATTGACAGACTGATAGATCTTTCATGATACAGTGATT

GGTGGTGCATGGCCGTTCTTAGTTGGTGGAGTGATCTGTCAGGTCTATTCCGGTAACGAG

CGAGACCACGATCTCTATTTTCTTTCAGTAGCATTTGTCGTTCTACTGAGAATAGAGAGA

CAACTAGTTCAAGCTAGGGGAAGCGTGGCAATAACAGGTCTGTGATGCCCTTCGATGTTC

TGGGCTGCACGCGCGCTACAATGGCAGCGACAAAAAGCATCACCTGCTCTGAGAAGAGTG

GGAAATCTTTAAAATCGCTGTCGTGATTGGGATTGAGCCTTGTAATAATTGCTCATGAAA

TAGGAATTCCTCGTAAGCGTGAGTCACCAACTCATGTTGAATACGTCTCTGCCCTTTGTA

CACACCGCCCGTCGCTAGTACCG

**28S** from GenBank

>FJ981818.1_C_shasta_28S

ACAATGATTCCCTTAGTAACTGCGAGTGAAGCGGGAAAAGATCAACGTTGAAATCTCAGT

GACTACGTTGCTGAGAGTTGTAACGTATAGACTATGTATCAAGTTTGTGTGTTGCTTTTA

AAATCTTCTGGATCGAAGTGCCATAGAGGGTGATAGCCCCGTAGTTAAAAGTGACATCAA

ATGCAAGATACATGGATCTTAGAGTCGGGTAATTTGGGATTGTTGCCTGAACCGAAGGTA

AACTCCTTCGAAATCTAAATATATTGTTTTTGACCGATAGCGAACAAGTACCGTGAGGGA

AAGGTGAAAAGAAATCTGTAAAGACAGTTAAAAGTACGTGAAACCGTTAACATGGAAGCT

GAGGGCAGTGTCGCGATTCATGGTATTAATTACAACTTGTGTCAAAGCAACGTTGTAATC

CGGTCCATGATGTTGTCAGAACCATTGACAATCTTCAAGGCCAGTGGAATAGCCTAGGAA

CTACAAATTAACAGGTTCTGCCTGCTGAAATGAATTCTTAGGTGTTCCTCACGCTGGTAC

GCAAGTTTAAACTTGAGAGTTGTTGTTGATGTTATTGAAATGTAAACTCTACTAGTTCCT

AGTTGAAACTAATGTAATTTTATGGTGCTGTTGTTATTACATGACGATAGTAGTGTATTT

TATGTTAGTTTTGAAGAGTTACGATTCTGACGAGATACCCATCTCTCAGACCCGTCTTGA

AACACGGACCAAGGAGGCAGAATCTAGAGCGAGTTGGAGGGTGTAAAACCCGACGGCGTA

GTGAAAGCAAAAGGTGTGATTCTGTTGAAAAATAGACGCAGCATCGGCCGGCCACATTTT

TAAATGTGATTGAGTTGGAGCTCTAGGTGCTGAACCCGAAAGATGGTGAACTATACGTGA

GCAGGGTAAAGCCAGAGGAAACTCTGGTGGAGGCTCGAAGTGATTCTGACGTGCAAATCG

ATCRTCAGACTTGCGTATAGGGGCGAAAGACTAATCGAACCATCTAGTAGCTGGTTCCCT

TCGAAATTTCCCTCAGGATCGCCGAGAATTTAATAGTTTCATCGGGTAGAGCAAATGATT

AGAGGAATCGGGGGAATGATTCCCTCGACCTATTCTCAAACTATCAACCGGTGAATGTAA

CAGTTTTGCATTGCTGAACTGTAACGTATTAATAAATATTCTCGAGTGGGCCATTTTTGG

TAAGCAGAACTGGCGATGCGGGATGAACCGAGAGGAGGGTTAAGGTGCCAAATACAGGTT

AATAGAGACCAGAAAAGGAGTCGGTCGATAAAGACAGCAGGACGGTGGCCCTGAAAGTAG

GAATCCGCTAAGGAGTGTCTAACAACTCACCTGCCGAATCGACGGGTCCTGAAAATGGAT

GGCGCTAAAGCCTGTAACCGATACCCTCCGTTCAGAGTGATATTTTGTTCTGAAAGGTAG

AAGGGCGCGGTGATTGTGTTAAAGTGTATAACGTGAGTATGCATGGAACGGTCACCGGTG

CGGATCTTGGTGTTAGTAGTGATGTATTAAAGCGAGAATCTTTAAGGCCGAAGTGGAGAA

GGGTTCCTCGTAAACAGTAGTTGGACGAGGGTTAGTCGGTCCTAAGTGGTTGGTTAATGC

TGTAAAATAGTTAACTAAGTAATTGGTTAAAACTCCACGAAAGGGAATCAGGTTAATATT

CCTGAACCAGATGTTGGAGATGTCATTAGCTTTTGTTAATGATAGAGACGGTAACGTAAG

CGAGCTCGCAGACGCTTTTAGCTAGTCCTGAGAGAATCATCTTTTCTGTATAACTGTTGA

ATGGCCTGGAATCGAGTCATTCGGAGAAGGGGCACGTAAACAGGAAGAGCACTGCTTTTG

GAGTGTCGGGATGTAGGTAATAGCCCTTGAAAATGCGAGCAAGCGAATGATTTTCACATC

TGTCCGTACCAAAACCGCATCAGGTCTCCAAGGTGAACAGCCTCTGGCATTGGAAGAATG

TGGGTAAGGGAAGTCGGCAAAATAGATCCGTAACTTCGGGAAAAGGATTGGCTCTAAGGA

CTGGGTTAATCGAGCTTTCCGCTGTAGGTGAAAAGGTAATCGAGCGGGTTGGCAACAATT

TGCGAGAGTATCGGTTTATTTACGTTGTGAGCTTGTTAATGTTTGTGGCAACATGAATGT

TAACTTAACGGTTAACGGTGATCAGTCAGCTTAGAACTGTTACTGACCAGGGGAATCCGA

CTGTTTAATTAAAACAAAGCATTCCGATGGCTGGAAACGGCGTTGACGGAATGTGATTTC

TGCCCAGTGCTCTGAATGTCAAAGTGTGGAAACGCAACCAAGCACGGGTCAACGGCGGGA

GTAACTATGACTCTCTAAAGGTAGCCAAATGCCTCGTCATCTAATTAGTGGCGCGCATGA

ATGGATCAACGAGATTCCCACTGTCCCTACCCATTATCCAACGAAAACACAGTCAAGGGA

ACGGGCTTGACTGAGACAGCGGGGAAAGAAGACCCTGTTGAGCTTGACTCTAGTTTGATT

TTGTGAGAAGACATGAGGGGTGTAGAATAGGTGGGAGAGCAGTTATGCTGTTCGACGTTG

AAATACCACTACTCTCATCGTTTTCTTACTTATTCGGTGCAAGGGGTAAGTGTTTGTAAG

TATAACTTACAAGCATGAAATTGGCCAAGCCAAGGCAGAGTAGATCTGTCGACTCTATTT

TTCGAAGACAGAATCAGATGGGGAGTTTGRCTGGGGCGGTACATCCTTAAAAATGCAACG

AGGATGTCCAAAGGCGAGCTCACCGTGGACAGAAACCACGGGTAGAGTAAAAGGGCAAAA

GCTCGCTTGATTCCGATTTTCAGTAAAAATACGAAACACGAAAGTGTGGCCTATCGATCC

TTTGACCATCCAGAGTTTGGAGTCAGAGGTGTCAGAAAAGTTACCACAGGGATAACTGGC

TTGTGGCGGCCAAGCGTTCATAGCGACGTCGCTTTTTGATCCTTCGATGTCGGCTCTTCC

TATCATTGGGTAGCAACTTGCCCAAAGCGTTGGATTGTTCACCCACTAACAGGGAACGTG

AGCTGGGTTTAGACCGTCGTGAGACAGGTTAGTTTTACCCTACTGTCTCTAAAGCACTGT

AACAGTAGTTCTGTTTAGTACGAGAGGACCTGCAGAACCGGATAAACGGTGAAGCATTTG

GTTGATAGGCCATAGATGCAAAGCTGTCATCCGCTG

**Supplementary material 2.** Average Cq values and their standard deviation indicating abundance of each gene in the sample. Average is calculated based on all biological replicates. Genes are listed according to their Cq values from low to high.

| **Organism** | **Genes** | **Blood stage** | **Sporogonic stage** |  |
| --- | --- | --- | --- | --- |
| *Sphaerospora molnari* | |  |  |  |
|  | 28S rRNA | 17.23 ± 4.39 | 16.09 ± 0.59 |  |
|  | 18S rRNA | 18.52 ± 4.47 | 17.08 ± 1.24 |  |
|  | ACTB | 23 ± 4.27 | 22.11 ± 0.63 |  |
|  | GAPDH | 28.78 ± 4.29 | 27.58 ± 0.38 |  |
|  | HPRT1 | 29.42 ± 3.7 | 26.4 ± 0.84 |  |
|  | EF2 | 28.94 ± 4.54 | 27.57 ± 0.47 |  |
|  | RPB2 | 33.78 ± 7.07 | 30.68 ± 2.1 |  |
|  | AHC1 | 37.73 ± 3.93 | 34.73 ± 0.93 |  |
| **Organism** | **Genes** | **Triactinomyxon stage** | **Sporogonic stage** |  |
| *Myxobolus cerebralis* |  |  |  |  |
|  | 18S rRNA | 14.9 ± 1.29 | 15.85 ± 2.52 |  |
|  | 28S rRNA | 20.77 ± 0.2 | 21.67 ± 1.64 |  |
|  | GAPDH | 26.12 ± 1.14 | 28.96 ± 2.89 |  |
|  | AHC1 | 28.44 ± 0.87 | 30.08 ± 3.8 |  |
|  | EF2 | 27.2 ± 0.76 | 31.2 ± 2.56 |  |
|  | ACTB | 28.15 ± 1.11 | 31.81 ± 2.68 |  |
|  | HPRT1 | 30.61 ± 1.4 | 34.77 ± 1.54 |  |
|  | RPB2 | 32.09 ± 1.22 | 36.12 ± 2.4 |  |
| **Organism** | **Genes** | **Tetractinomyxon stage** | **Presporogonic & sporogonic stages** | |
| *Ceratonova shasta* |  |  | **Ascites** | **Intestine** |
|  | 28S rRNA | 19.97 ±1.0 | 15.22 ±0.27 | 18.97 ±0.01 |
|  | 18S rRNA | 25.67 ±1.34 | 18.09 ±1.54 | 23.9 ±2.62 |
|  | ACTB | 30.17 ±2.45 | 20.31 ±1.16 | 28.96 ±5.29 |
|  | GAPDH | 31.37 ±2.78 | 23.54 ±1.83 | 28.56 ±3.19 |
|  | AHC1 | 32.8 ±1.1 | 23.65 ±1.81 | 30.31 ±4.07 |
|  | EF2 | 33.17 ±0.2 | 25.93 ±0.0 | 32.41 ±2.13 |
|  | RPB2 | 42.21 ±2.23 | 37.3 ±0.47 | 43.52 ±2.55 |
|  |  |  |  |  |

**Supplementary material 3.** Example of melting curves of eight candidate genes in *Sphaerospora molnari* blood and sporogonic stages (for biological replicate 1), demonstrating PCR primer specificity.


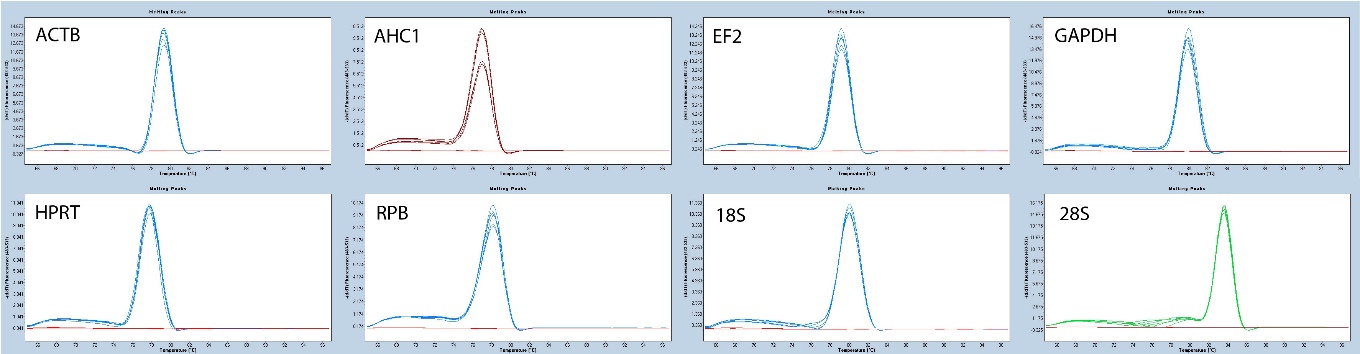


**Supplementary material 4.** Stability values obtained by different algorithms supporting Gene stability ranking presented in Table 5.

| ***Sphaerospora molnari*** | |  |  |  |  |
| --- | --- | --- | --- | --- | --- |
| **Genes** | **geNorm M** | **NormFinder Stability value** | **BestKeeper SD ± Cq** | Δ *Cq* Average of STDEV | **RefFinder Geomean of ranking values** |
| ACTB | 0.4 | 0.51 | 2.13 | 1.17 | 2.28 |
| AHC1 | 0.99 | 1.17 | 2.58 | 1.53 | 5.73 |
| EF2 | 0.37 | 0.18 | 2.34 | 1.08 | 1.41 |
| GAPDH | 0.37 | 0.38 | 2.15 | 1.12 | 1.68 |
| HPRT1 | 1.11 | 1.35 | 2.42 | 1.64 | 6.44 |
| RPB2 | 1.48 | 2.46 | 3.94 | 2.6 | 8 |
| 18S rRNA | 0.77 | 1.13 | 2.6 | 1.55 | 5.69 |
| 28S rRNA | 0.54 | 0.58 | 2.28 | 1.21 | 3.72 |
|  |  |  |  |  |  |
| ***Myxobolus cerebralis*** | | |  |  |  |
| **Genes** | **geNorm M** | **NormFinder Stability value** | **BestKeeper SD ± Cq** | Δ *Cq* Average of STDEV | **RefFinder Geomean of ranking values** |
| ACTB | 0.41 | 0.56 | 1.95 | 1.12 | 2.11 |
| AHC1 | 1.09 | 1.22 | 1.54 | 1.57 | 4.82 |
| EF2 | 0.41 | 0.65 | 2 | 1.17 | 2.71 |
| GAPDH | 0.57 | 0.27 | 1.69 | 1.11 | 1.86 |
| HPRT1 | 0.84 | 1.27 | 2.21 | 1.51 | 5.88 |
| RPB2 | 0.73 | 0.96 | 2.17 | 1.3 | 4.6 |
| 18S rRNA | 1.4 | 1.49 | 1.34 | 1.74 | 5.65 |
| 28S rRNA | 1.29 | 1.38 | 0.7 | 1.67 | 4.3 |
|  |  |  |  |  |  |
| ***Ceratonova shasta*** |  |  |  |  |  |
| **Genes** | **geNorm M** | **NormFinder Stability value** | **BestKeeper SD ± Cq** | Δ *Cq* Average of STDEV | **RefFinder Geomean of ranking values** |
| ACTB | 1.27 | 2 | 3.65 | 2.89 | 2.99 |
| AHC1 | 1.27 | 1.92 | 3.34 | 2.86 | 2.44 |
| EF2 | 1.94 | 0.94 | 3.06 | 2.79 | 2.21 |
| GAPDH | 1.54 | 1.15 | 2.87 | 2.63 | 1.86 |
| HPRT1 | Excluded from analysis | |  |  |  |
| RPB2 | Excluded from analysis | |  |  |  |
| 18S rRNA | 3.26 | 4.6 | 4.28 | 4.89 | 6 |
| 28S rRNA | 2.44 | 2.5 | 1.97 | 3.48 | 3.34 |
|  |  |  |  |  |  |

**Supplementary material 5. Notes to methodological challenges**

RNA extraction and genomic DNA removal from the RNA samples

Total host+parasite RNA was isolated using the Nucleospin RNA Kit (Macherey-Nagel) following manufacturer's instructions. A DNase digestion step ensuring elimination of genomic DNA was included into the protocol of the Nucleospin RNA Kit. We obtained adequate amount of RNA concentration (30-400 ng/µl) for all the samples with exception of *Manayunkia* worms that in the best case hardly would reach to 6-7 ng/µl. We assumed that Nucleospin RNA Kit (Macherey-Nagel) is not suitable for such type of small worms, since it is based on column-based purification approach, and most probably the few amount of RNA remained on the column membrane. We have tried to isolate *Manayunkia* worms using classical guanidine/thiocyanate/phenol/chloroform based extraction method ^46^, which allowed to obtain 4 to 5 times higher concentration compared to the column-based RNA extraction methods. The higher concentrations were probably due to presence of genomic DNA in the sample, since guanidine/thiocyanate/phenol/chloroform based-extraction method does not include DNase digestion step. For *Manayunkia* samples genomic DNA was digested using DNAFree Kit (Invitrogen) following by manufacturer's instructions. After genomic DNA digestion step, the concentrations of RNA in *Manayunkia* samples dropped 50 %, however it was still higher compared to extraction with Nucleospin RNA Kit (Macherey-Nagel) kit (resulting in average 10-30 ng/µl). RNA concentration and purity were checked using a Nano Drop - 1000 Spectrophotometer (Thermo Fisher Scientific Inc.). All the RNA samples with 260/280 ratio in range of 1.9-2.0, and 260/230 ratio in range of 2.0-2.4 were chosen for cDNA synthesis. Approximately 500 ng RNA was used as an input for 20 µl of final volume cDNA synthesis using the Transcriptor High Fidelity cDNA synthesis Kit (Roche) following the manufacturer's protocol.

Primer design and PCR specificity

Gene-specific primers were designed to amplify short 70-150 bp regions suitable for RT-qPCR assays (Table 3). Primer pairs were designed with optimal Tm at 58-60°C and GC content between 45-50%, using the NCBI online primer-design tool <https://www.ncbi.nlm.nih.gov/tools/primer-blast/>. All primers were tested for specificity using conventional PCR prior to performing RT-qPCR. The following conditions were used for conventional PCR: 95°C for 5 min; 28 cycles of denaturation at 95°C for 1 min, annealing at 58°C for 1 min, elongation 68°C for 1 min; and final elongation of 68°C for 8 min. PCRs were conducted in 10 µl volume using the following reagents: Titanium Taq DNA Polymerase 0.5 Unit per 10 µl (Takara Bio Europe/Clontech, SaintGermain en Laye, France) PCR Buffer 1X (Clontech), 200 µM dNTP mix (Promega, USA), 0.5 µM forward and reverse primer, ~ 200 ng input cDNA (a pool of host-parasite cDNA from the infected tissue) and PCR grade water (variable). Primer specificity was determined by obtaining single amplicons of the expected size from infected samples and no amplification in uninfected fish samples (indicating that primers are not annealing with fish cDNA). Controls without reverse transcriptase (–RT) were tested for genomic DNA contamination. The presence of infection, observed microscopically when taking the samples, was confirmed by the presence of visible bands on 1% agarose gel in TAE buffer. Bands were isolated from gel and purified using the Gel/PCR Fragment Extraction Kit (Geneaid Biotech Ltd., New Taipei City, Taiwan). PCR products were commercially sequenced using Sanger sequencing (Seqme, Czech Republic, www.SEQme.eu) and their identity was confirmed by sequence comparison. Primer specificity was also checked by running melting curve analysis.

PCR efficiency

While RT-qPCR is considered one of the most reliable techniques to accurately measure the expression level of a gene, primer efficiency can become a real obstacle to obtain accurate data. PCR efficiency can be influenced by PCR inhibitors present in the sample or by non-optimal primer design. This information is critical, since these factors can produce different results even if the experimental design or study organism is similar. While acceptable optimum range for primer efficiency is considered 90-110%, in reality efficiencies often slide out of these borders.

We have obtained PCR efficiency from 88-129% (see table below), which is slightly passing the borders of acceptable efficiency range. However, we obtained similar efficiencies for the given genes in two different developmental stages of parasite, thus it did not affect our calculations. Overall, even if our primers efficiency values are not perfectly fitting to the optimal ranges of efficiency, we believe that they can be still acceptable giving the challenging nature of our samples and study.

PCR inhibitors are one of the most common cause for non-efficient PCR. We have used inhibitor removal columns (QIagen, E.Z.N.A.), as well as different concentrations of Bovine Serum Albumin (50 ng, 500 ng, 1250 ng conc.) to reduce inhibitory effect, however this did not improve our overall results. Besides, column based inhibitory removal methods are not suitable, when one has to deal with small amount of sample. While serial dilutions can help to deal with inhibition, this was not useful in our case. When parasite is intermixed with host, it is difficult to evaluate which is the true concentration of parasite in the sample, and very often the amount of parasite is extremely low. This can be a challenge, since the cDNA can be totally lost after few steps of dilution.

Besides presence of inhibitors, poor primer design also could be a reason for non-optimal efficiency. We have designed and tested several primers before choosing final list of the primers used in this study.

| **Organism** | **Gene** | **Primer sequence (5'-3')** | **Efficiency %** | |
| --- | --- | --- | --- | --- |
|  |  |  | **blood** | **gills** |
| *Sphaerospora molnari* | ACTB | F: AATCCACGAGACCACCTTCG | 119 | 121 |
|  |  | R: CAGCAGCCAAACCGGTGATA |  |  |
|  | AHC1 | F: TTCCCCATGGTGTCGAGAAA | 88 | 87.9 |
|  |  | R: TCAATGACACCTCGAACACAGT |  |  |
|  | EF2 | F: TCCGGCAGGCAAGAAGGTTT | 100 | 99 |
|  |  | R: CCAAGTTGGATACGGATTACGAGT |  |  |
|  | GAPDH | F: TATCGACCTGGCCGTTACTG | 100 | 100 |
|  |  | R: GTTGCTGCTGTCAATGACCC |  |  |
|  | HPRT 1 | F: TCTCATCTGTGACCGTGCTC | 88 | 89 |
|  |  | R: ACGCACAAAAACTCGGATCTG |  |  |
|  | RPB2 | F: ATTAGTTACGGTGCCGGAGG | 129 | 121 |
|  |  | R: GCTGTGACATGGAAGATGCG |  |  |
|  | 18S rRNA | F: ATCCCAGGTCGTATCCGCTA | 99 | 100 |
|  |  | R: ACTGCCCTGTTGATGCGATT |  |  |
|  | 28S rRNA | F: ATCTGCTCGCACCTCATACG | 119 | 119 |
|  |  | R: CCGAGTTTGCTTGCGTTACC |  |  |
| *Myxobolus cerebralis* |  |  | fish cartilage | worm |
|  | ACTB | F: TTGCCTGATGGTCAGGTGAT |  |  |
|  |  | R: AGTGTCTCGTGAAGTCCACTG | 93 | 89.3 |
|  | AHC1 | F: GTTCAGCGTCGCTAAGAGGA | 129 | 121 |
|  |  | R: GCCCGAGAGACACAGTCATC |  |  |
|  | EF2 | F: ATGGATCCGGGCCTAACCTT | 116.8 | 119 |
|  |  | R: CAAGTCCAGACGAACACCCC |  |  |
|  | GAPDH | F: GTGGCAAAACCCGCAACTAA |  |  |
|  |  | R: TGTGCGTCGACAAACTGGAT | 105.8 | 105.1 |
|  | HPRT 1 | F: TGGTGCTCCTGGTGAAGAAA |  |  |
|  |  | R: GAGGTCGTCCATCCCAGTTT | 80.6 | 81.2 |
|  | RPB2 | F: AATGGAGGGCTGGCTAAACG |  |  |
|  |  | R: TAATCCGATGTCAGGGCACC | 87.1 | 88 |
|  | 18S rRNA | F: TAGAGTGTGCCGAACGAGTC |  |  |
|  |  | R: GGTCCCAAGGCATCATGACA | 93 | 99 |
|  | 28S rRNA | F: AGTCGAAGTAGAGCAGCGTG | 99 | 99 |
|  |  | R: CATCCTCAGGGATGCACTGT |  |  |
| *Ceratonova shasta* |  |  | fish intestine | worm |
|  | ACTB | F: GTCGGCAATTCCTGGGTACA | 93.7 |  |
|  |  | R: TCCAACCGGCATTTTTAGGA |  |  |
|  | AHC1 | F: TTCGGTTACCACGACTCGGC | 100 |  |
|  |  | R: TGTAGTGGGTGGCTATGGTGA |  |  |
|  | EF2 | F: CTGGATTCCAATGGGCAACT | 91 |  |
|  |  | R: AAATAACTCTTCGAGCAGTAGGT |  |  |
|  | GAPDH | F: TGGGGCTAAACAGTTGGTGG |  |  |
|  |  | R: GTGGACATTTGAAAGGAGGCG | 121 |  |
|  | RPB2 | F: TGGAGGTTGAAGGTACGTGT | 100 |  |
|  |  | R: TCTGCCCCTTTATAGGACGA |  |  |
|  | 18S rRNA | F: CCAAGTTGGTCTCTCCGTGA | 88 |  |
|  |  | R: CAAATTAAGCCGCAGGCTCC |  |  |
|  | 28S rRNA | F: ACGTGAAACCGTTAACATGGA | 119 |  |
|  |  | R: CCACTGGCCTTGAAGATTGT |  |  |
